# Evaluation of the Global Performance of Eight *In Silico* Skin Sensitization Models Using Human Data

**DOI:** 10.14573/altex.1911261

**Published:** 2020-05-07

**Authors:** Emily Golden, Donna S. Macmillan, Greg Dameron, Petra Kern, Thomas Hartung, Alexandra Maertens

**Affiliations:** 1 Center for Alternatives to Animal Testing (CAAT), Johns Hopkins Bloomberg School of Public Health, Baltimore, MD, USA;; 2 Lhasa Limited, Leeds, UK;; 3 Procter & Gamble NV, Bever, Belgium;; 4 CAAT-Europe, University of Konstanz, Konstanz, Germany

## Abstract

Allergic contact dermatitis, or the clinical manifestation of skin sensitization, is a leading occupational hazard. Several testing approaches exist to assess skin sensitization, but *in silico* models are perhaps the most advantageous due to their high speed and low-cost results. Many *in silico* skin sensitization models exist, though many have only been tested against results from animal studies (e.g., LLNA); this creates uncertainty in human skin sensitization assessments in both a screening and regulatory context. This project’s aim was to evaluate the accuracy of eight *in silico* skin sensitization models against two human data sets: one highly curated (Basketter et al., 2014) and one screening level (HSDB). The binary skin sensitization status of each chemical in each of the two data sets was compared to the prediction from eight *in silico* skin sensitization tools (Toxtree, PredSkin, OECD’s QSAR Toolbox, UL’s REACHAcross^™^, Danish QSAR Database, TIMES-SS, and Lhasa Limited’s Derek Nexus). Models were assessed for coverage, accuracy, sensitivity, and specificity, as well as optimization features (e.g., probability of accuracy, applicability domain, etc.), if available. While there was a wide range of sensitivity and specificity, the models generally performed comparably to the LLNA in predicting human skin sensitization status (i.e., approximately 70–80% accuracy). Additionally, the models did not mispredict the same compounds, suggesting there might be an advantage in combining models. *In silico* skin sensitization models offer accurate and useful insights in a screening context; however, further improvements are necessary so these models may be considered fully reliable for regulatory applications.

## 1 Introduction

Skin sensitization is defined as an allergic response that follows chemical contact with the skin (UN, 2017). For most sensitizers, an electrophilic chemical must penetrate the skin and bind to proteins, which activates cellular immune responses and results in immunological priming. Upon re-exposure to the chemical, an allergic response can follow (Kimber et al., 2002). These events and responses have collectively been identified as the adverse outcome pathway (AOP) for skin sensitization (OECD, 2012). Metals and metal salts also can be sensitizers, although possibly through a different mechanism (Schmidt et al., 2010).

In humans, skin sensitization, which is more commonly referred to as allergic contact dermatitis, has been reported to affect approximately 20% of the population (Thyssen et al., 2007), making it a major human health concern, especially in occupational settings (Cashman et al., 2012; Kadivar and Belsito, 2015; Warshaw et al., 2017). Traditionally, the skin sensitization potential of chemicals has been assessed using animal methods, such as the guinea pig maximization test (GPMT) and the Buehler assay. The mouse local lymph node assay (LLNA) has been validated as a refined animal model to evaluate skin sensitization (Sailstad et al., 2001).

The utilization of these animal methods has recently been met with several challenges. First and foremost, in 2013, the EU finalized its complete ban on animal testing for hazards of ingredients in cosmetics marketed in the EU (EU, 2009; EC, 2016). Existing animal methods are also costly and require a substantial amount of time to carry out (NRC, 2007). Further, the relevance of animal studies to humans has repeatedly been called into question (NRC, 2007; Hartung, 2008, 2009; Leist and Hartung, 2013; Pound and Bracken, 2014; Bailey et al., 2014, 2015; Akhtar, 2015). This is a burden to regulators and manufacturers alike, as the combination of increased restrictions on animal testing as well as the high cost and lengthy turnaround time of traditional animal methods makes them increasingly unsuitable for chemical hazard assessment.

Because of increased concerns about animal welfare on the part of both consumers and regulatory agencies, there has been increased emphasis on developing non-animal test methods to predict skin sensitization as an alternative to the LLNA (Basketter et al., 2012). Several types of non-animal alternative methods have been developed to address these issues, including a) tests for direct chemical and biochemical reactivity (*in chemico*), b) tests for effects in cultured cells, tissues, and organs (*in vitro*), and c) computer-modeled prediction of hazards (*in silico*). The direct peptide reactivity assay (DPRA) is an *in chemico* method used to predict the reactivity of the target chemical with skin proteins, which is the molecular initiating event (MIE) in the skin sensitization AOP (OECD, 2019). Validated *in vitro* models include the ARE-NrF2 luciferase test method (KeratinoSens^™^ and LuSens) (OECD, 2018a), which measures the activation of keratinocytes, the second key event in the AOP. The human cell line activation test (h-CLAT), the U937 cell line activation test (U-SENS^™^), and the interleukin-8 reporter gene assay (IL-8) (OECD, 2018b) are also validated *in vitro* methods that evaluate activation of dendritic cells – the third key event in the AOP for skin sensitization. Available *in silico* models are numerous (Wilm et al., 2018; Alves et al., 2018) and include those that assess structural alerts (Enoch et al., 2011), use a read-across approach (Hartung, 2016; Luechtefeld and Hartung, 2017), or employ quantitative structure activity relationships ((Q)SARs) (Chaudhry et al., 2010; Alves et al., 2016a). Finally, combinations of these tools can be used to capture the various steps in the skin sensitization AOP in what is referred to as a defined approach (DA) (OECD, 2016). Most DAs are hazard-based schemes that may include sequential testing strategies (STS) or integrated testing strategies (ITS) (Hartung et al., 2013). As the names imply, the STS is a stepwise approach in which the user can make a hazard prediction at any point in the strategy, while an ITS employs all steps before a hazard prediction is made.

*In chemico* and *in vitro* models provide valuable information; however, these methods are restricted to laboratories, which reduces their convenience. DAs are a relatively new concept, and, while they are gaining momentum due to improved accuracy over traditional approaches to evaluate skin sensitization (Kleinstreuer et al., 2018), only a limited number of well-described ITS are publicly available (Johansson and Lindstedt, 2014; Luechtefeld et al., 2015; Jaworska, 2016; Macmillan and Chilton, 2019). *In silico* models are generally less expensive and faster (Raunio, 2011) and can be implemented almost ubiquitously, unlike their laboratory-based counterparts. This makes them attractive tools for hazard evaluation, particularly in a screening context, as chemical and product manufacturers seek methods to quickly and cost-effectively identify skin-sensitizing chemicals to protect both workers and consumers (Cronin, 2010; Cronin and Madden, 2010; Maertens et al., 2014; Maertens and Hartung, 2018).

Due to ethical considerations, publicly available skin sensitization data in humans are generally limited (Basketter, 2009); therefore, the development of *in silico* skin sensitization models has relied heavily on data from animal studies, as well as *in vitro* studies. Numerous reviews have evaluated the performance of *in silico* skin sensitization models against animal data (Chaudry et al., 2010; Bauch et al., 2012; Teubner et al., 2013; Kostal and Vouchkova-Kostal, 2016; Verheyen et al., 2017; Fitzpatrick et al., 2018); however, less consideration has been given to how *in silico* models predict skin sensitization against human data (Bauch et al., 2012; Verheyen et al., 2017; Luechtefeld et al., 2018a). This analysis aims to: (i) assess the availability and quality of human skin sensitization data for hazard screening assessments, and (ii) identify strengths and weaknesses of current *in silico* skin sensitization models so that they may be properly applied and improved for future use in human hazard assessment.

## 2 Methods

### Data sets

A set of 131 chemicals and their corresponding human skin sensitization statuses compiled by Basketter et al. (2014) were used in this assessment (see Tab. S1^1^). The skin sensitization statuses assigned in Basketter et al. (2014) were largely based on human predictive and diagnostic patch test data, and the potencies ranged from Human Category 1 to Human Category 6, with chemicals in Category 1 being the most potent sensitizers and chemicals in Category 5 being weak sensitizers; chemicals in Category 6 were defined as non-sensitizers. For this analysis, the categories were converted to binary outcomes (i.e., “sensitizer” or “non-sensitizer”), i.e., Category 1 through 5 were converted to sensitizers, whereas Category 6 chemicals were converted to non-sensitizers. See Basketter et al. (2014) for additional details on the definitions of the initial assignment of Categories 1 through 6.

The Chemical Abstract Services Registry Numbers (CASRNs) were taken from Basketter et al. (2014), and the Simplified Molecular Input Line Entry System (SMILES) strings were obtained from ChemIDplus Advanced^2^. No SMILES string was available in ChemIDplus Advanced for 8 of the 131 chemicals in the Basketter et al. (2014) data set; however, in order to control for SMILES string variation, only ChemIDplus Advanced was used as a source for SMILES strings. Nevertheless, these 8 chemicals were still included in the data set because some of the models can utilize CASRN as an input.

Additionally, a manually curated human skin sensitization data set of 375 chemicals was prepared by querying the Hazardous Substances Data Bank (HSDB) for chemicals associated with the search term “skin sensitization”^3^ (see Tab. S2^1^). HSDB is a peer-reviewed, toxicological database supplemented with information such as human and environmental exposures, industrial hygiene, and regulatory requirements. HSDB data are reported as study summaries, warning statements, or other abbreviated hazard records. It was selected as a literature source due to its large, centralized collection of human data and relevance to occupational exposures.

“Skin sensitization” was used as the search term in the HSDB to identify human skin sensitization data. This search produced 923 discrete chemical records. Each occurrence of “skin sensitization” or “sensitization” in each record was reviewed; this led to the review of 1,264 summaries (some chemical records had multiple occurrences of the terms “sensitization” or “skin sensitization”). In HSDB, summaries of various information sources, rather than complete dossiers, data sheets, etc., are provided. Information sources summarized include case reports, drug warnings, epidemiology studies, human exposure studies, safety data sheets, etc. If it was not clear from the summary whether a skin sensitization study was exclusively based on human data, it was not included in the final data set. This review process eliminated 411 chemicals, as these contained no human data, leaving 512 chemicals with human data. In order to have a discrete identifier, chemicals without a CASRN were not included in the final data set, which eliminated 13 of the 512 chemicals, leaving 499 chemicals with human data and a CASRN.

Finally, from these 499 chemicals, 124 chemicals were eliminated due to incomplete or unclear summaries. Incomplete or unclear summaries were those that were considered to have insufficient information to characterize the skin sensitization status of the chemical. For example, if route of exposure was not clearly given as dermal in the summary, the chemical was not included in the final data set. Additionally, if a chemical appeared to be administered as part of a mixture, it was not included in the final data set. This led to a final data set of 375 chemicals with human skin sensitization data on which the sensitization status of a chemical could be based. Chemicals were considered to be skin sensitizers if there was evidence of sensitization; consequently, the chemicals in this data set likely capture a range of sensitization potency. Chemicals were considered to be non-sensitizers if the authors concluded no sensitization reaction was observed. The CASRNs were taken from the HSDB entry for each chemical, and the SMILES strings were again obtained solely from ChemIDplus Advanced^2^. Of the 375 chemicals in the HSDB data set, SMILES strings were unavailable for 14 chemicals. Again, these 14 chemicals remained in the data set, as some models are capable of using the CASRN as an input.

The Basketter et al. (2014) chemical data set represents a data set that was culled from the literature and critically reviewed by experts; in contradistinction, the HSDB data set represents the quality and availability of data typically used when carrying out a chemical hazard screening assessment and is not as precise in distinguishing skin sensitization potency.

### CLP comparison

To assess the concordance of our skin sensitization statuses assigned to the Basketter et al. (2014) and HSDB data sets with the more standardized approach adopted in the Classification, Labelling, and Packaging (CLP) Regulation ((EC) No 1272/2008), we compared our data with the harmonized skin sensitization classifications as assigned according to the CLP (ECHA, 2018a). The CLP Regulation requires chemical manufacturers to classify the hazard of their product, communicate this hazard through appropriate labeling, and package the product in a manner that addresses the hazards. To date, the European Chemicals Agency (ECHA) has assigned hazard classifications for several thousand chemicals (i.e., harmonized classifications), and the remaining chemicals available in commerce must be self-classified (ECHA, 2018b).

### Model performance

The skin sensitization status of all chemicals from both data sets was then predicted via 8 *in silico* models using either the CASRN or the SMILES string for each chemical, depending on the required input of each model. An overview of each model is summarized in Table 1.

The chemicals from the two data sets were evaluated in batch mode for all models, with the exception of REACHAcross^™^ (Luechtefeld et al., 2018b): The version used in this analysis does not offer a batch mode, so the chemicals were processed individually using CASRNs. It should be noted that some of the chemicals have structures with multi-position substituents; consequently, their SMILES strings are unspecified. Some of the models that use SMILES strings as the input parameter (i.e., QSAR Toolbox, CAESAR, TIMES-SS, and Derek Nexus (Derek)) have a strict SMILES string interpretation, and, as a result, some of the ambiguous SMILES strings in the data set were not predicted.

The global performance of all models was evaluated by assessing the following parameters: coverage, sensitivity, specificity, false predictions, accuracy, and balanced accuracy. For false predictions, the chemical class, partition coefficient, and molecular weight were evaluated to identify patterns of false predictions (if any) in the models. The chemical class for each chemical was assigned using the U.S. EPA New Chemical Category from QSAR Toolbox. The octanol:water partition coefficients were identified using the KOWWIN model in U.S. EPA’s EPISuite, and the molecular weights were obtained from ChemIDplus Advanced^2^.

Some of the models offer additional information about the prediction. PredSkin and REACHAcross^™^ offer an associated probability of accuracy with the returned prediction, and the distribution of these probabilities was assessed to evaluate the confidence in the predictions provided by the models. Additionally, TIMES-SS is highly dependent on metabolism, so a separate assessment of the effect of metabolism on prediction accuracy was included in this evaluation.

Finally, there is some debate as to whether the Human Category 5 chemicals (i.e., rare sensitizers) from the Basketter et al. (2014) data set should be considered sensitizers. Skin sensitization has been reported following exposure to these chemicals; however, the data available are not sufficient to classify these chemicals as skin sensitizers according to GHS (see Basketter et al., 2014 for additional details). Therefore, a separate exploration was performed in this analysis in which these chemicals were considered non-sensitizers, and the effect on model accuracy was evaluated.

## 3 Results

### 3.1 Data sets

The Basketter et al. (2014) data set consists of 131 chemicals, of which approximately 80% are sensitizers representing a wide range of potencies and 20% are non-sensitizers (see Tab. 2). In the HSDB data set (n = 375 chemicals), approximately 60% of the chemicals are sensitizers and 40% are non-sensitizers.

**Fig. 1:**
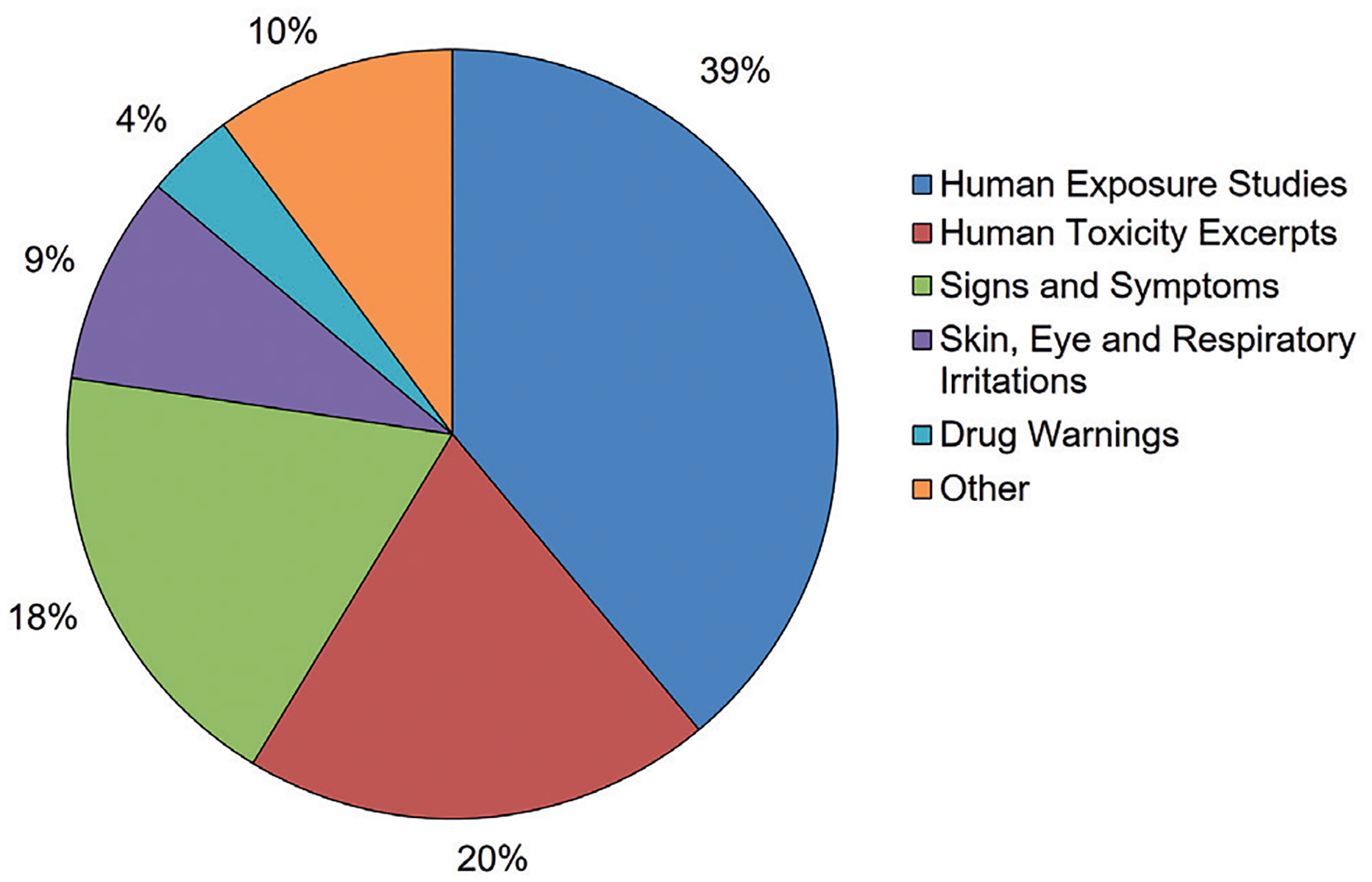
Distribution of the HSDB entries by data source for all chemicals in the final HSDB data set The HSDB entries summarize skin sensitization studies in humans and categorize them according to source type. Those source types that accounted for ≤ 1% of all source types were combined in “Other” for clarity.

Chemical summaries in the final HSDB data set were drawn from roughly 16 source types; however, 80% of the skin sensitization summaries were extracted from only three source types: Human Exposure Studies, Human Toxicity Excerpts, and Signs and Symptoms. The distribution of all data sources is provided in Figure 1.

**Fig. 2:**
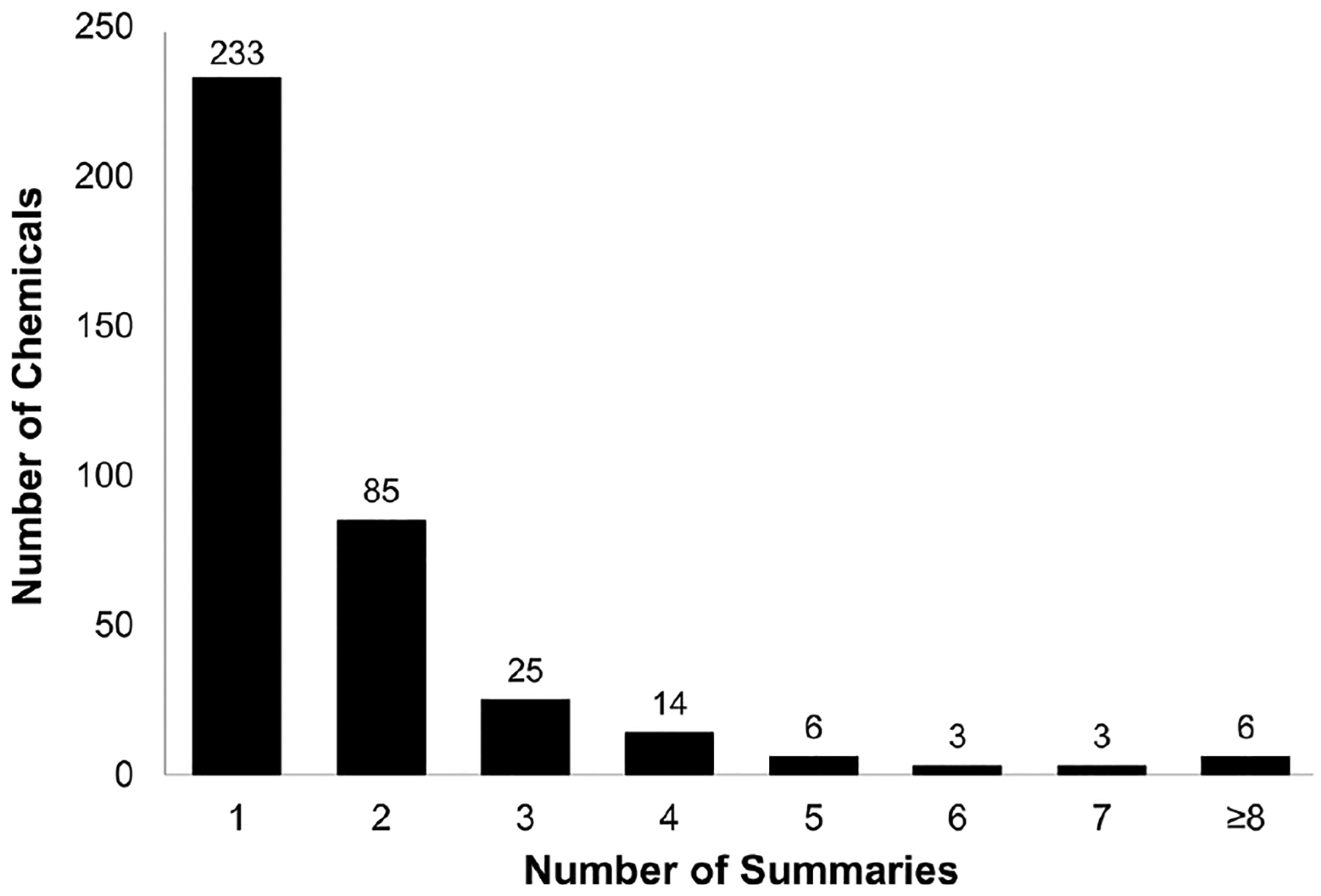
Distribution of the number of human skin sensitization summaries across chemicals in the HSDB data set

Hazard data availability for the HSDB data set is summarized in Figure 2. For over 60% of the chemicals in the final HSDB data set, only one summary was available. Overall, there were few chemicals with multiple summaries: only 18 chemicals had 5 summaries or more available.

### 3.2 CLP comparison

Less than half of the chemicals in each of the data sets had harmonized skin sensitization classifications in the CLP, as illustrated in Table 3. When the CLP harmonized classifications were compared to classifications in Basketter et al. (2014), the concordance was 78%, which was higher than the concordance of the CLP classifications compared to the HSDB data set at 64%. Basketter et al. (2014) had a similar percent concordant positive (100% and 92%, respectively) and percent concordant negative (45% and 41%, respectively) to HSDB. The HSDB data set had a discordant positive rate of 59%, which was also very similar to Basketter et al. (2014) at 55%. No discordant negatives were assigned in the Basketter et al. (2014) data set, and a low number of discordant negatives were assigned in the HSDB data set (8%).

### 3.3 Data set comparison

Of the 131 and 375 chemicals assessed in Basketter et al. (2014) and HSDB, respectively, only 8% of the chemicals (n = 41) occurred in both data sets. The concordance for all chemicals with overlap was 78%. The binary concordance between the Basketter et al. (2014) and HSDB data sets are shown by potency category, as assigned in Basketter et al. (2014), in Table 4. The chemicals that were in both data sets had relatively high concordance for most potency categories, with concordance ranging from 86% to 100%. The only exception to this was for those chemicals that were classified by Basketter et al. (2014) as rare sensitizers (Human Category 5); these chemicals had low concordance (40%).

### 3.4 Model performance

Model performance was assessed based on model coverage, accuracy, balanced accuracy, sensitivity, and specificity. Base model settings were defined as the prediction returned by simply entering the input parameter (i.e., CASRN or SMILES) into the model; this was considered the *base result*. To better illustrate the capabilities of each model, optimal model settings were also used to assess all performance measures, and the prediction produced by incorporating these optimal settings was the *high confidence result*. The optimization features included applicability domain, probability of accuracy, and metabolism, if available. Only high confidence results are presented for the model performance metrics except for coverage; both base and high confidence results are presented for coverage. Optimization settings (i.e., the settings applied to obtain a high confidence result) for each model are shown in Table 5.

For PredSkin and REACHAcross^™^, a high confidence result was one that fell within the applicability domain and had a probability of accuracy greater than 70%. For QSAR Toolbox, the prediction had to be within the applicability domain; both the base and optimized settings for QSAR Toolbox have the same coverage because the automated batch mode applies the same settings universally. For the Danish QSAR Database, CAESAR, and TIMES-SS, a high confidence result was defined as a prediction falling within the applicability domain. A high confidence result for Derek was defined as every prediction with the exception of “non-sensitizer with misclassified and/or unclassified features.” Toxtree is a structural alert model and, therefore, does not have any optimization features, so the model output was considered to be the high confidence result.

Of note, the TIMES-SS model provides predictions in potency format; however, for this assessment, the results were converted to binary format. Additionally, Derek provides likelihood levels for alerting chemicals, which were converted to binary format (certain, probable, plausible, equivocal = sensitizer; doubted, improbable, impossible = non-sensitizer) for this assessment. REACHAcross^™^ provides results in both binary and GHS Category format; for this assessment, the binary results were used. QSAR Toolbox has the potential to predict skin potency values (i.e., EC3 values) in the automated workflow; however, the binary results from the automated workflow were used for this assessment. The use of binary results in the context of this assessment allowed for easier comparison of global performance across all models.

**Fig. 3:**
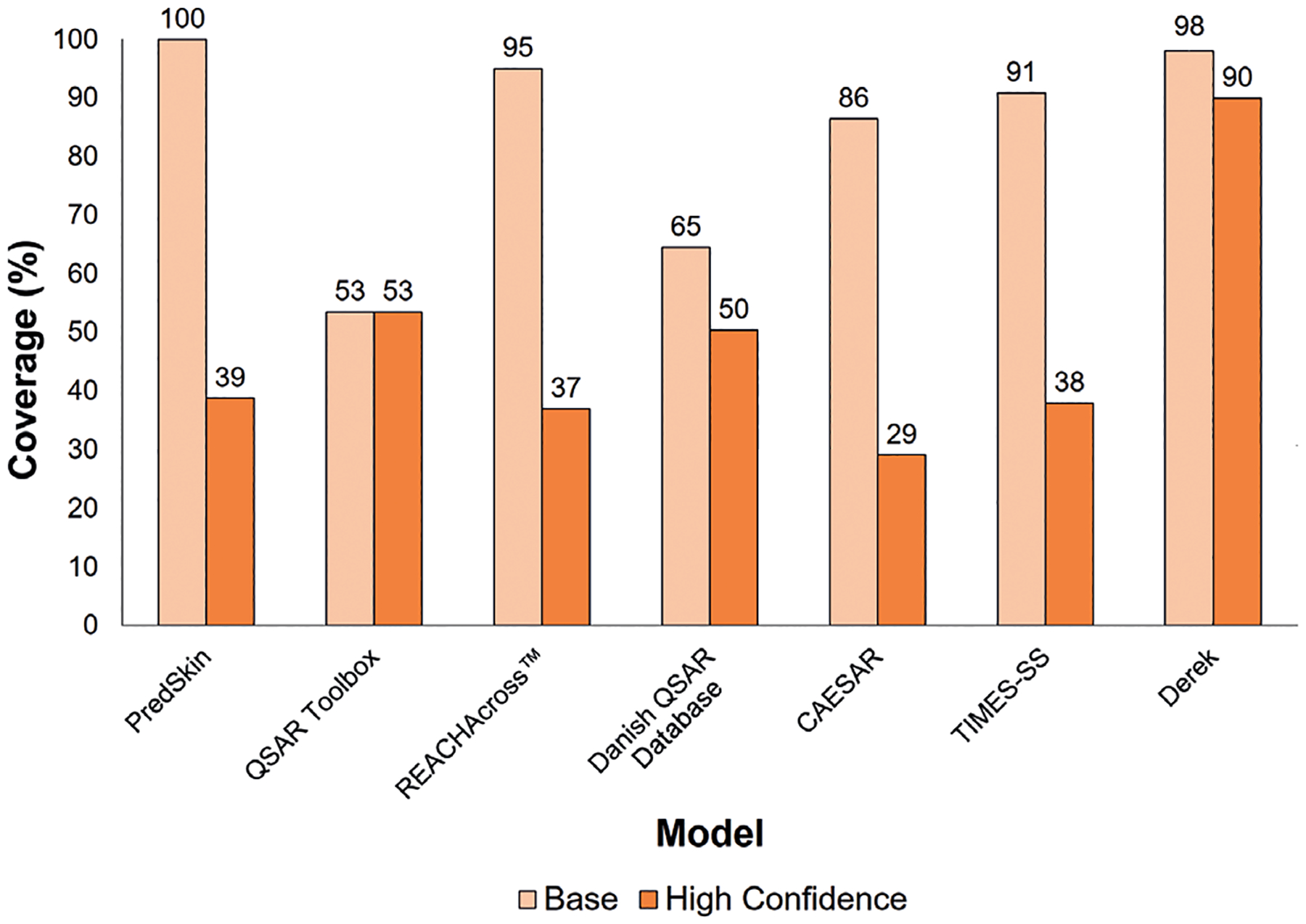
Base and high confidence model coverage for HSDB data set Base results (calculated using base settings) and high confidence results (calculated using optimized settings) for model coverage calculated by evaluating the number of returned predictions over the total number of chemicals with model input parameters. Base results were calculated by assessing the number of predictions produced without applying optimization features. High confidence results were obtained by implementing optimization features. Toxtree does not have any optimization features; consequently, the model coverage, which is 100% under both conditions due to its structural alert nature, is not shown here.

Model coverage for base and optimized settings using the HSDB data set is illustrated in Figure 3. Using base settings, the model coverages were similar for several of the models: PredSkin, REACHAcross^™^, CAESAR, TIMES-SS, and Derek all had base coverages above 85%. The Danish QSAR Database had a base model coverage of 65%, while the QSAR Toolbox had a base coverage of 53%. However, it should be noted that the QSAR Toolbox coverage was the same under base and optimized conditions because the same settings are always applied for the automated batch method used in this model. For the optimized settings, Derek had the greatest model coverage at 90% for the HSDB data set. The other models produced coverages ranging from 30% to approximately 50%. The model coverages for the Basketter et al. (2014) data set (both base and optimal settings) can be found in Figure S1^1^, and the number of chemicals entered into each model and the number of predictions returned for both data sets can be found in Table S3^1^.

**Fig. 4:**
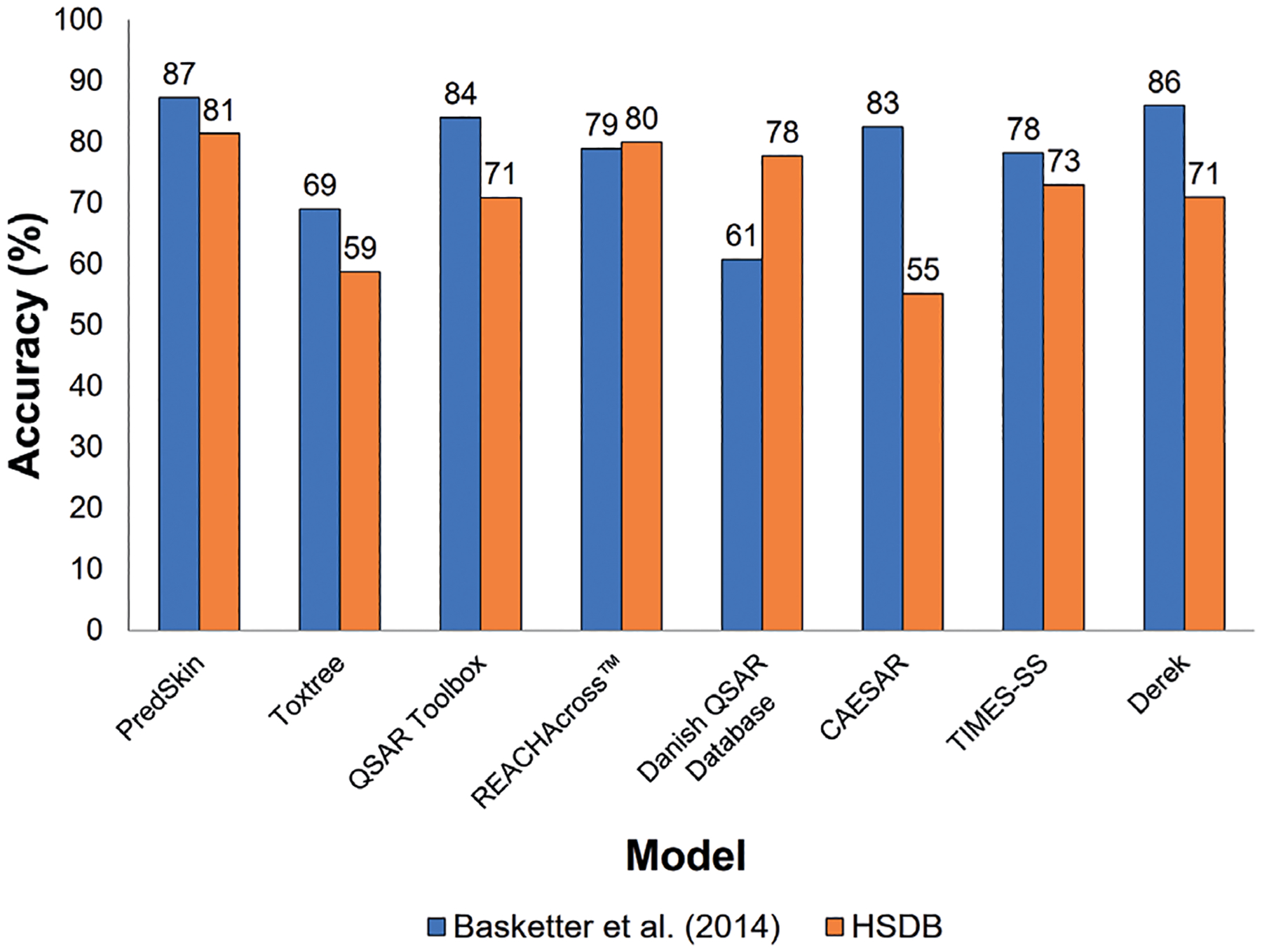
Model accuracy for high confidence results For the Basketter et al. (2014) data set, model accuracy was calculated by assessing concordance between the sensitization status assigned in Basketter et al. (2014) and the skin sensitization prediction (using optimized settings) from each model. For the HSDB data set, model accuracy was calculated by assessing concordance between the HSDB skin sensitization status and the skin sensitization prediction (using optimized settings) from each model. Only chemicals with a returned prediction were included in the accuracy assessments.

Accuracy and balanced accuracy both had wide ranges across the models; however, the majority of the models tended to perform comparably, as demonstrated by the similarity in accuracy and balanced accuracy for many of the models. As shown in Figure 4, the accuracy range of the models for both the Basketter et al. (2014) and HSDB data sets was 26%. Overall, PredSkin had the highest accuracy for the Basketter et al. (2014) data set at 87%, but several of the other models also achieved similar accuracy, including Derek (86%), QSAR Toolbox (84%), CAESAR (83%), REACHAcross^™^ (79%), and TIMES-SS (78%). Danish QSAR Database had the lowest accuracy for the Basketter et al. (2014) data set at 61%. For the HSDB data set, PredSkin had the highest accuracy at 81%, but, again, many of the other models – REACHAcross^™^ (80%), Danish QSAR Database (78%), TIMES-SS (73%), QSAR Toolbox (71%), and Derek (71%) – were within 10%. CAESAR had the lowest accuracy for the HSDB data set at 55%.

**Fig. 5:**
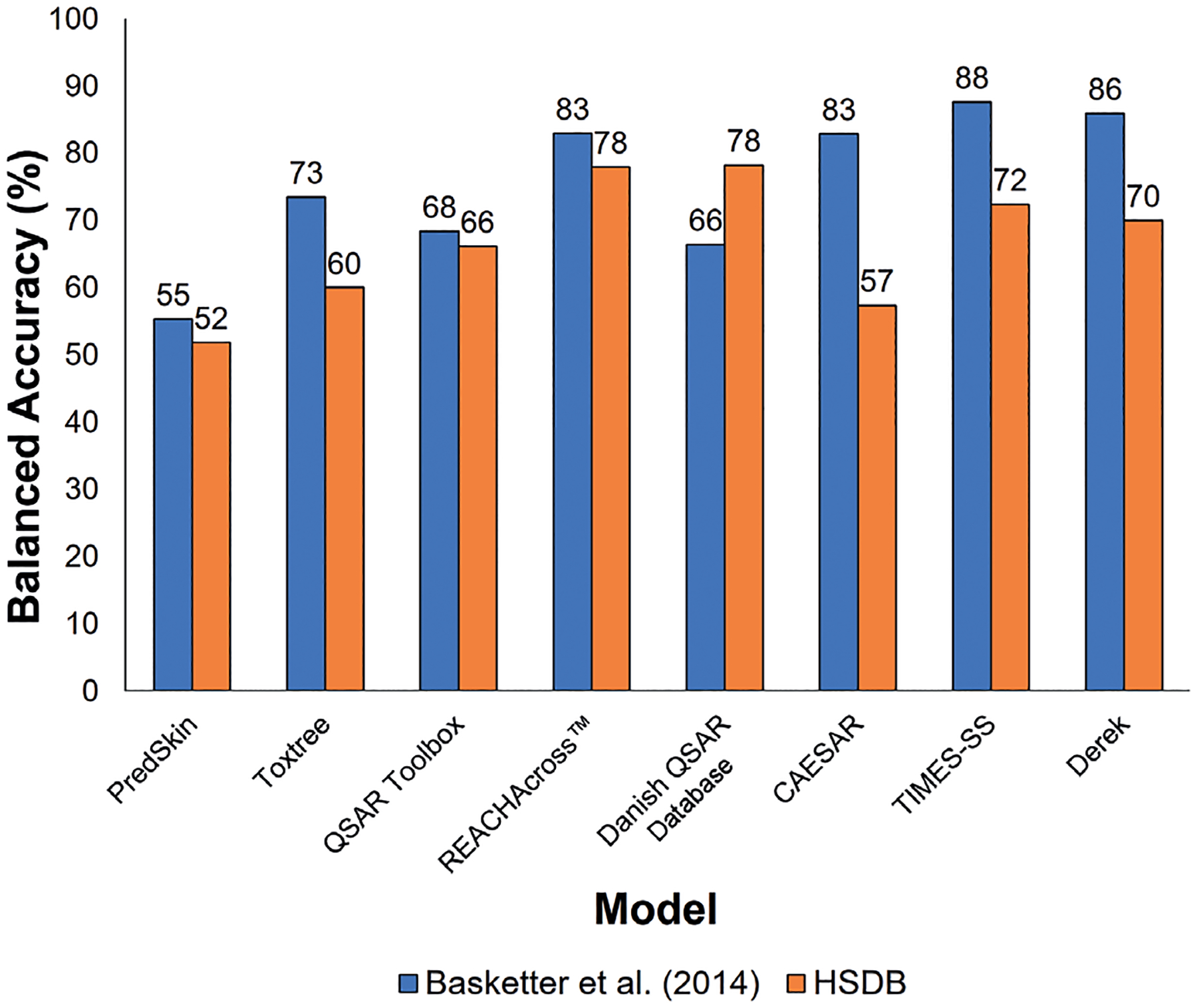
Model balanced accuracy for high confidence results For the Basketter et al. (2014) data set, balanced accuracy was calculated by assessing concordance between the sensitization status assigned in Basketter et al. (2014) and the skin sensitization prediction from each of the models and accounting for the unbalanced distribution of sensitizing and non-sensitizing chemicals in the data set. The same procedure was carried out for calculation of balanced accuracy for the HSDB data set. Only chemicals with a returned prediction were included in the balanced accuracy assessment.

For both data sets, there were more sensitizers than non-sensitizers, and, therefore, the data set is unbalanced; as a result, balanced accuracy (Fig. 5) is a better metric for model performance. The balanced accuracy range of the models following evaluation of the Basketter et al. (2014) data set was 33% while the balanced accuracy range of the models after evaluation of the HSDB data set was 26%. Overall, TIMES-SS had the highest balanced accuracy for the Basketter et al. (2014) data set at 88%, but, as with model accuracy, other models had balanced accuracies that were comparable, including Derek (86%), REACHAcross^™^ (83%), and CAESAR (83%). PredSkin had the lowest balanced accuracy for the Basketter et al. (2014) data set at 55%.

For the HSDB data set, REACHAcross^™^ and the Danish QSAR Database both had the highest balanced accuracy at 78%, but TIMES-SS and Derek had similar balanced accuracies at 72% and 70%, respectively. PredSkin, again, had the lowest balanced accuracy at 52%.

**Fig. 6:**
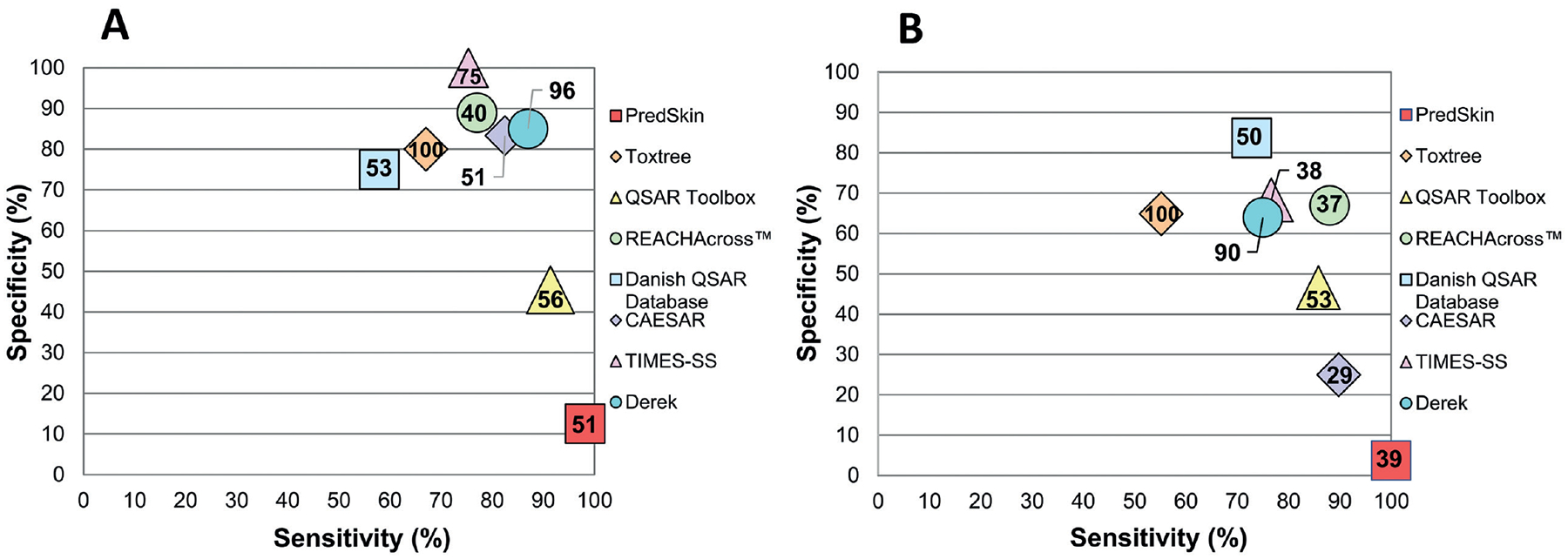
Model specificity, sensitivity, and coverage for Basketter et al. (2014) and HSDB data sets for high confidence results Model specificity and sensitivity for each model against (A) the Basketter et al. (2014) data set and (B) the HSDB data set. In this figure, specificity and sensitivity were calculated based on high confidence results, and the values in the data points represent the model coverage (%) based on high confidence results.

With the exception of Danish QSAR Database and Toxtree, which had fairly low sensitivity, the majority of models exhibited similar sensitivities (75%−90%); PredSkin had the highest sensitivity at 98% for the Basketter et al. (2014) data set (see Fig. 6A). Model specificity had a much wider range (87%) for the Basketter et al. (2014) data set. TIMES-SS had perfect specificity at 100%, while PredSkin had the lowest specificity at 13%. QSAR Toolbox also had relatively low specificity at 45%, while the remaining models had specificities ranging from approximately 70% to almost 90%. Figure 6A presents the sensitivity and specificity of each model as well as model coverage (indicated by the number inside each symbol). The specific percentages for sensitivity and specificity can be found in Table S4^1^, and the false prediction percentages for each model can be found in Table S5^1^.

The range of model sensitivity for the HSDB data set was 45%. PredSkin, again, achieved the highest sensitivity at 100% while Toxtree had the lowest sensitivity at 55%. The sensitivities of the remaining models were moderate to high, ranging from approximately 70% to nearly 90%.

Like the Basketter et al. (2014) data set, model specificity for the HSDB data set had a much wider range than sensitivity at 80%. The Danish QSAR Database had the highest specificity at 84% while PredSkin, again, had the lowest specificity at 4%. CAESAR and, again, QSAR Toolbox had relatively low specificity at 25% and 47%, respectively, while the other models had specificities ranging from 64% to 68%. The sensitivity and specificity of each model for the HSDB data set is depicted in Figure 6B, along with the model coverages (indicated by the values within each symbol). The specific percentages for sensitivity and specificity can be found in Table S4^1^, and the false prediction percentages for each model can be found in Table S5^1^.

**Fig. 7:**
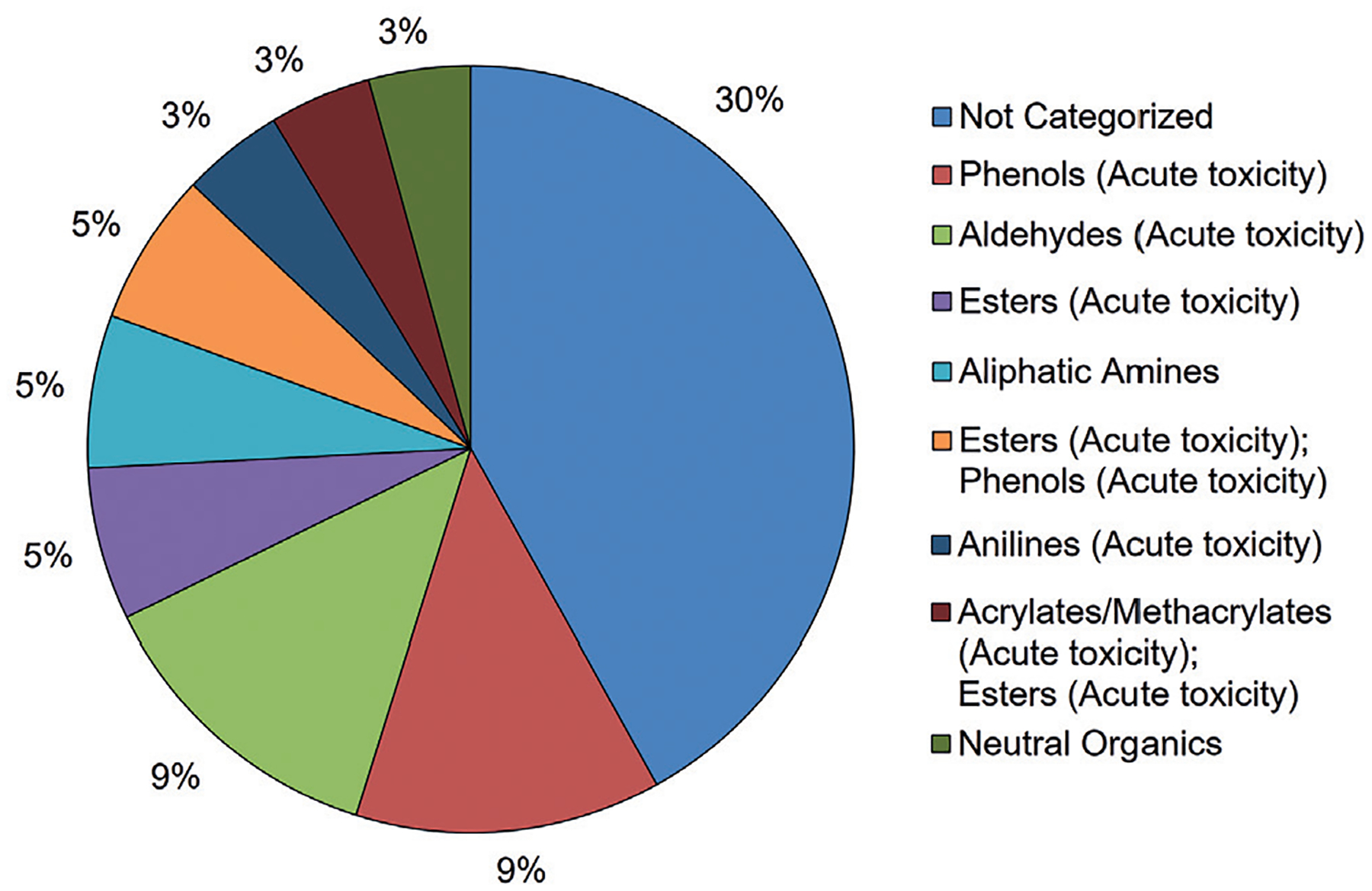
Distribution by U.S. EPA New Chemicals Category for the Basketter et al. (2014) data set Chemical categories for the Basketter et al. (2014) data set (n = 131) were assigned using the U.S. EPA New Chemicals Categorization tool in the QSAR Toolbox. If more than one functional group is identified in a category, it is because the chemical has both functional groups. Chemical categories that account for < 3% of the total chemical category distribution are not shown in the figure for clarity.

To determine if there were any patterns in incorrect predictions, mispredictions were assessed by chemical category, partition coefficient, and molecular weight. The chemical category (assigned using the U.S. EPA New Chemical Category database in QSAR Toolbox) distribution for the Basketter et al. (2014) data set is shown in Figure 7. A total of 28 chemical categories were identified for the Basketter et al. (2014) data set. The majority of chemicals were classified as “Not Categorized”, meaning that they did not meet the chemical definitions of any chemical category as defined under the U.S. EPA New Chemicals Program. Chemical categories that occurred with high frequency in the Basketter et al. (2014) data set were phenols, aldehydes, esters, aliphatic amines, and ester/phenols.

**Fig. 8:**
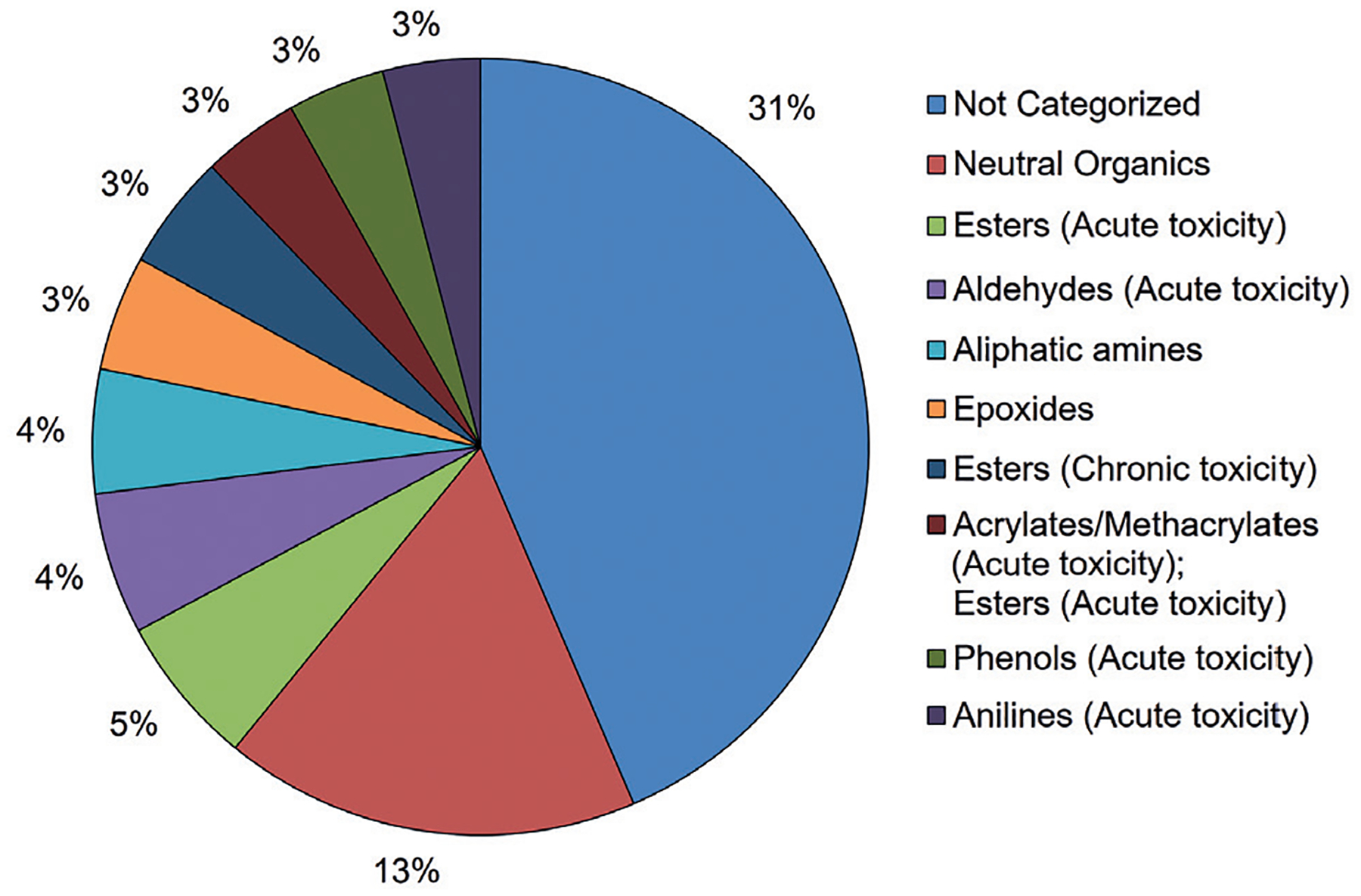
Distribution by U.S. EPA New Chemicals Category for the HSDB data set Chemical categories for the HSDB data set (n = 375) were assigned using the U.S. EPA New Chemicals Categorization tool in the QSAR Toolbox. If more than one functional group is identified in a category, it is because the chemical has both functional groups. Chemical categories that account for < 3% of the total chemical category distribution are not shown in the figure for clarity.

The HSDB data set incorporated a larger variety of chemical categories (n = 45). The distribution of these categories is shown in Figure 8. Again, the majority of chemicals were classified as “Not Categorized”. Neutral organics, esters, aldehydes, and aliphatic amines were among the chemical categories that occurred with the highest frequencies in the HSDB data set.

**Fig. 9:**
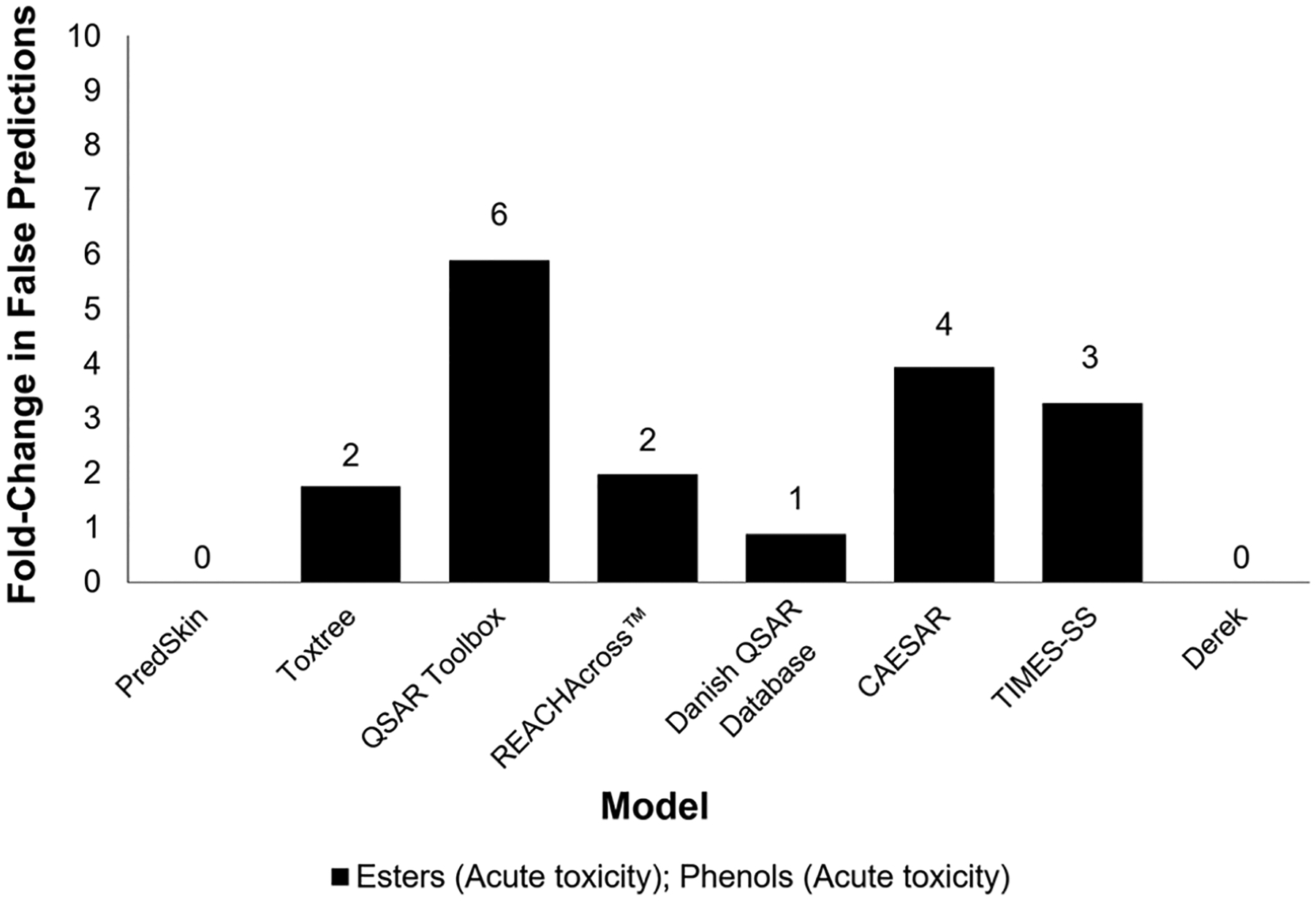
False prediction analysis for Basketter et al. (2014) data set for high confidence results This figure presents the fold-change in false predictions for the “esters (acute toxicity); phenols (acute toxicity)” U.S. EPA New Chemicals Category (i.e., chemicals that contain both an ester and phenol functional group). False predictions from each model were compared to the expected false predictions in the overall distribution in the Basketter et al. (2014) data set to obtain the fold-change in false predictions.

Mispredictions were assessed by chemical category to determine if they were falsely predicted at an increased frequency due to issues with the models assessing certain chemical categories or simply by chance. In general, the frequency of false predictions was not increased when compared to the expected distribution for either data set, with the exception of the ester/phenol chemical class in the Basketter et al. (2014) data set (see Fig. 9), which had an increased incidence of false predictions in many of the models, including QSAR Toolbox (6-fold increase), CAESAR (4-fold increase), TIMES-SS (3-fold increase), REACHAcross^™^ (2-fold increase), and Toxtree (2-fold increase). For the HSDB data set, the frequency of false predictions was not increased for any chemical category.

It has often been assumed that above a certain molecular weight or partition coefficient, a chemical is not a skin sensitizer (Smith Pease et al., 2003). Our data set contained few chemicals that were above the molecular weight of 500 g/mol or with a log K_OW_ ≥ 5 (Fig. S2–S5^1^), so we did not perform a sub-analysis of those features. However, more robust analysis of larger data sets has demonstrated that the molecular weight cut-off reduces the probability that a chemical is a skin sensitizer but does not preclude it (Gerberick et al., 2005; Fitzpatrick et al., 2016; Luechtefeld et al., 2016).

**Fig. 10:**
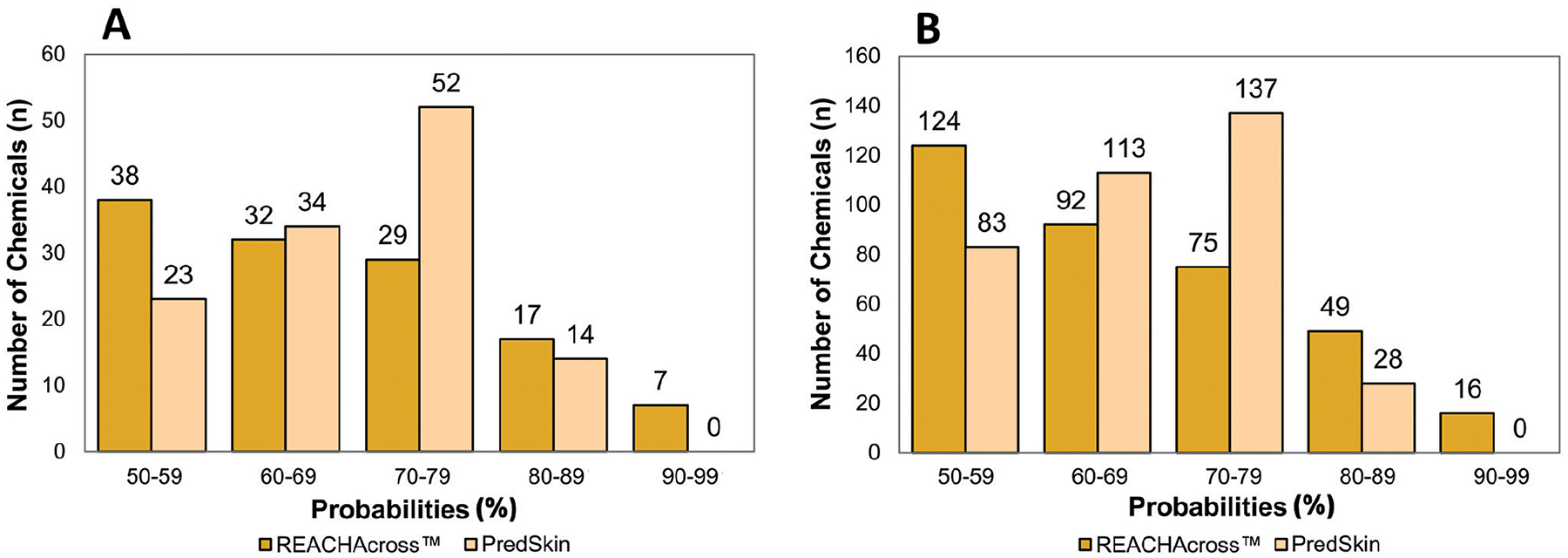
Probability distribution for PredSkin and REACHAcross^™^ for Basketter et al. (2014) and HSDB data sets Both REACHAcross and PredSkin provide a probability to assess confidence in the prediction – both PredSkin and REACHAcross^™^ had relatively few predictions above the 80 percent threshold for A) the Basketter et al. (2014) data set and B) the HSDB data set.

Two of the models include a probability of the accuracy of the returned prediction: PredSkin and REACHAcross^™^. These models each provide predictions with a probability of accuracy down to 50%. The distributions of probabilities for both models for the Basketter et al. (2014) and the HSDB data sets are shown in Figure 10A and 10B, respectively. REACHAcross^™^ had some predictions that had very high probabilities of accuracy (i.e., 90–99% range) for both data sets, but the majority of the predictions for REACHAcross^™^ were associated with 50–59% probability of accuracy for both data sets. The highest probability of accuracy for a prediction from PredSkin fell in the 80–89% range for both data sets, but the majority of the predictions were associated with a probability of accuracy between 70 and 79%.

While PredSkin and REACHAcross^™^ offer a probability of accuracy feature, TIMES-SS and QSAR Toolbox both include a metabolism feature in their design. QSAR Toolbox considers metabolism in its automated workflow skin sensitization assessment; however, in Version 4.1 of the QSAR Toolbox (the version used in this analysis), the automated workflow in batch mode does not identify whether the parent or metabolite is the chemical on which the skin sensitization alert is based. As a result, QSAR Toolbox was not included in this metabolism sub-analysis.

TIMES-SS is designed to perform optimally when metabolism is incorporated. TIMES-SS evaluates the skin sensitization potential of the parent compound and all potential metabolites. If the parent compound is a sensitizer, then the chemical is predicted to be a sensitizer. If the parent compound is not predicted to be a sensitizer but the metabolite is predicted to be a sensitizer, the overall sensitization status is predicted to be a sensitizer based on the predicted status of the metabolite. If neither the parent chemical nor the metabolite is predicted to be a skin sensitizer, then the overall prediction is “non-sensitizer”.

**Fig. 11:**
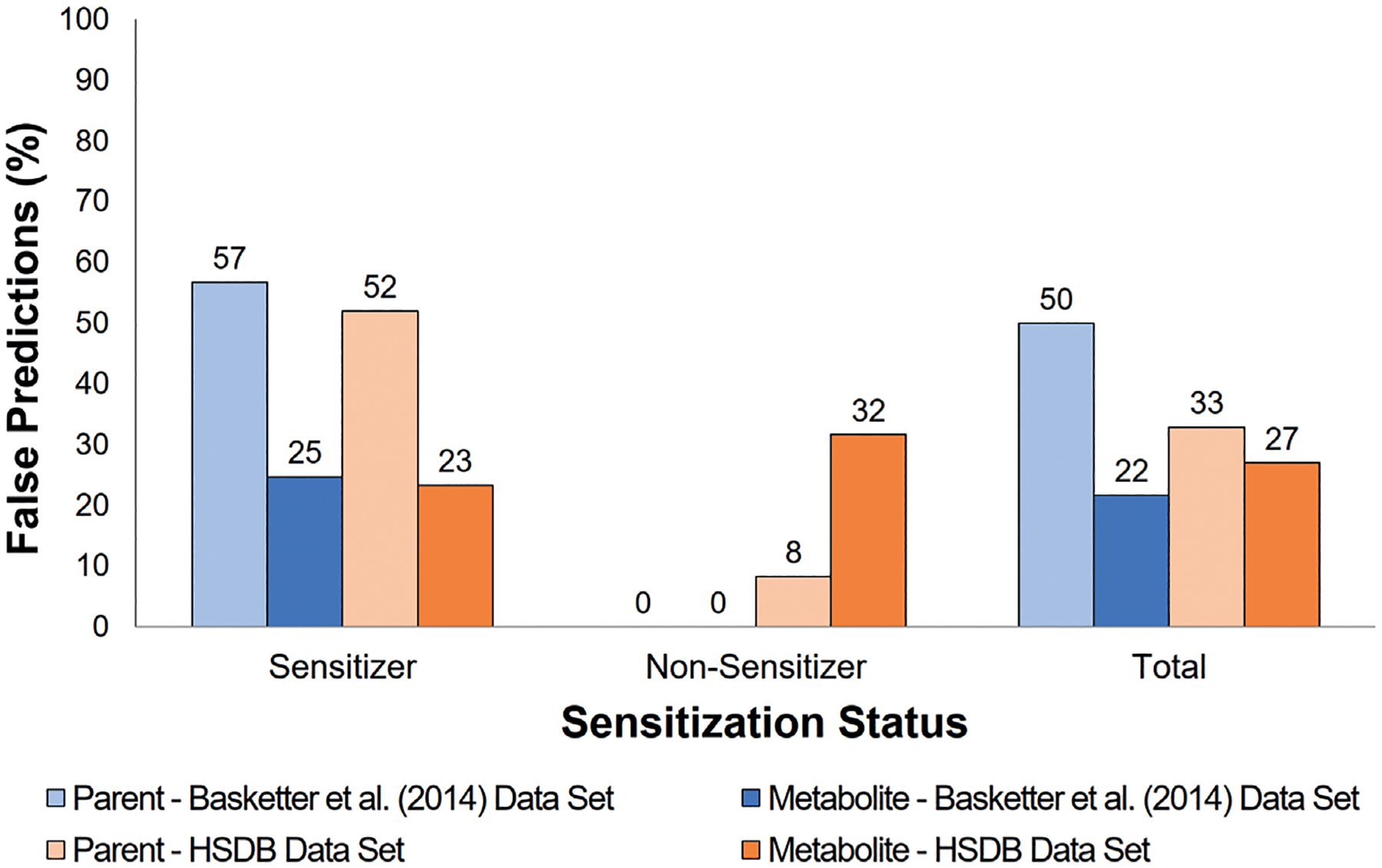
Effect of metabolism on false predictions Effect of metabolism on model false predictions using the TIMES-SS model for the Basketter et al. (2014) and HSDB data sets. Results that consider the prediction for parent compounds only are shown in pale bars while results that consider predictions for metabolites (where applicable) are shown in bright bars.

The effect of metabolism on accuracy is shown in Figure 11. Overall model accuracy improved significantly in the Basketter et al. (2014) data set and modestly in the HSDB data set after incorporation of metabolism. This was particularly striking for sensitizers, as the accuracy increased by approximately 30% in both data sets. However, metabolism did not appear to affect accuracy for non-sensitizers in the Basketter et al. (2014) data set, and it decreased the accuracy of non-sensitizers in the HSDB data set. Likewise, total false predictions decreased substantially for the Basketter et al. (2014) data set and modestly for the HSDB data set after consideration of metabolism. Again, predictions for sensitizers improved most when metabolism was incorporated, as false predictions decreased around 30% for both data sets. However, the false predictions for the non-sensitizers were unaffected by metabolism in the Basketter et al. (2014) data set and increased for the HSDB data set.

An analysis to determine the effect of the sensitization status of Category 5 chemicals in the Basketter et al. (2014) data set on model accuracy was performed. Specifically, the sensitization status of the Category 5 chemicals was considered to be negative for the purposes of this part of the assessment (in previous parts of this assessment, they were categorized as positive for sensitization). The model accuracies and balanced accuracies were recalculated to determine how model accuracy was affected by this change in skin sensitization status. The results of the model accuracies and balanced accuracies with Category 5 as a positive skin sensitization category (i.e., the sensitization status originally assigned to the Basketter et al. (2014) data set) and with Category 5 as a negative skin sensitization category are compared in Table 6. As shown, some model accuracies and balanced accuracies improved while others declined; there was no clear pattern in the effect of Category 5 skin sensitization status in the model accuracies and balanced accuracies.

## 4 Discussion

### 4.1 HSDB data set

While the curated data set from HSDB offers a fairly large and novel data set for human hazard assessment, it also illustrates the lack of depth and precision typically available to hazard assessors, especially for human data. To begin with, terminology is often confused: during the curation process, it was observed that the literature contains numerous other terms that are intended to represent skin sensitization, including contact dermatitis, contact eczema, eczematous dermatosis, and skin allergy. Skin sensitization is defined as a delayed hypersensitivity reaction (Roberts and Patlewicz, 2009), but some of these terms refer to conditions with immediate reactions. Further, some terms are umbrella terms meant to capture several skin conditions (e.g., contact dermatitis is an umbrella term that may refer to allergic or irritant contact dermatitis, and irritant contact dermatitis is not an allergic response (Cashman et al., 2012)) and may not specifically represent a delayed sensitization reaction. Therefore, the lack of precision in reporting human health effects both makes data curation more difficult and compromises data accuracy. One key necessity for improving our ability to predict human skin sensitizers will be a more concerted effort to use accurate and consistent terminology in reporting human skin sensitization data.

The most frequently occurring category was Human Exposure Studies; summaries from this category provided a relatively high level of detail, including test substance description and protocol information (e.g., number of test subjects, dose, etc.). While entries from this HSDB category comprised studies that were relatively well-described, many of the chemical entries for other high-volume categories, such as Signs and Symptoms, reported only hazard classifications from regulatory documents such as Safety Data Sheets (SDS) or International Chemical Safety Cards (ICSC), or brief statements from summary texts, such as chemical or toxicity handbooks (e.g., Pohanish, 2012; Dreisbach, 1977; Hayes and Laws, 1991). Due to the limited details provided from studies/statements drawn from the Signs and Symptoms category, it often was not clear whether the primary skin sensitization data from these sources was from human or animal data. Some summaries also failed to provide sufficient details on the identity of the test substance, which made it unclear whether the test substance in the HSDB summaries was a pure chemical or a mixture, limiting the usefulness of the studies. Consequently, test substance data must be reported in a transparent manner to ensure accurate hazard assessment. Moreover, given that most chemicals in the data set had only one reliable study and/or hazard statement on which the skin sensitization status of the chemical was based, this highlights the need for increased data availability, especially for human data. Nonetheless, even limited data of lower quality can serve as an informative starting point for skin sensitization characterization, especially in a screening level hazard assessment.

It is noteworthy that, with the exception of the Danish QSAR Database, the accuracies and balanced accuracies for all the models were greater for the Basketter et al. (2014) data set than for the HSDB data set. It is likely that many of the chemicals in the Basketter et al. (2014) data set are in the training set for many, if not all, of these models. The HSDB data set includes a wider range of chemical classes and, arguably, provides a more realistic picture of the chemical space encountered in hazard assessment.

### 4.2 Model performance

Model performance was evaluated on several metrics, including coverage, accuracy/balanced accuracy, sensitivity, specificity, and false prediction rates. Because one performance metric cannot necessarily be weighted higher than the others and because the models incorporate optimization features to different degrees, it is not possible to select one model that clearly outperforms the others. Still, some of the models offer advantages over the others.

First, even a simple structural alert model like Toxtree performs comparably to more complicated read-across and QSAR models. Compared to the balanced accuracies for the read-across and QSAR models, Toxtree ranked in the middle of all the other models’ balanced accuracies for both the Basketter et al. (2014) and HSDB data sets. This implies that structural alerts are still useful in the evaluation of skin sensitization. However, they should be used with caution, as Toxtree had one of the highest percentages for false negatives for the Basketter et al. (2014) data set at 33% and had the highest number of false negatives for the HSDB data set at 45%, which suggests that it is not as conservative in its predictions as read-across and QSAR models. On the other hand, by virtue of being a structural alert system, Toxtree had complete coverage of both data sets and therefore serves as a useful first step in a screening hazard assessment to prioritize chemicals through either additional modeling and/or testing. In fact, the screening/confirmatory approach as well as combining structural alerts with read-across models and/or QSAR models has been evaluated and proposed in other work (Alves et al., 2016b). Similarly, Derek, another structural alert-based model, which pairs structural alerts with expert rules, illustrates the power of a structural-alert model, as supported by its high coverage and low incidence of false predictions. Specifically, this model has the potential to resolve false predictions by applying expert rules that address common structural alerts which trigger false predictions. This pairing allows for increased accuracy when compared to a basic structural alert model.

Additionally, TIMES-SS saw considerable improvement in prediction accuracy for sensitizers and a decrease in the false predictions in both the Basketter et al. (2014) and HSDB data sets when metabolism was incorporated, emphasizing the relevance of metabolism to skin sensitization (Smith Pease et al., 2003). However, there was an increase in the false positives rate for non-sensitizers in the HSDB with predicted metabolism. Thus, the effects of metabolism predictions need to be better understood for reaching conclusions for non-sensitizers, perhaps in combination with a better understanding of skin penetration. Other models that do not explicitly address metabolism through skin metabolism simulators, as TIMES-SS does, still have relatively high accuracy, although this may be because metabolism is implicitly included through the inclusion of chemicals that require metabolic activation in the training data sets and classification based on *in vivo* studies where metabolism naturally occurs. However, metabolism should be implemented with caution to avoid overly-conservative hazard assessments. Therefore, if metabolism is predicted to drive the skin sensitization status of a chemical, it should be flagged for further review to determine whether the metabolite is both feasible and relevant.

Very few *in silico* tools offer a probability of accuracy, but this feature can improve model performance if employed correctly. Predskin and REACHAcross^™^ both offer this “optimization” feature, but it comes with strengths and weaknesses. PredSkin and REACHAcross^™^ both make predictions where data availability is minimal; however, this results in some predictions with low probability of accuracy, with some predictions returning a probability of accuracy as low as 50%. Because a probability of accuracy this low is not sufficient to confidently determine the sensitization status of a chemical, it is a near impossible case to make for regulatory acceptance. However, the transparency provided by the probability of accuracy is useful to the hazard assessor: It informs the confidence of the prediction, allowing the hazard assessor to more accurately determine whether the prediction is worth incorporating into a chemical hazard assessment. It should be noted that Derek also offers a probability of accuracy metric (likelihood levels); however, as it is a qualitative metric, it was not included in this analysis.

QSAR Toolbox’s accuracy highlights the importance of two factors in the evaluation of skin sensitization: selection of good analogs and consideration of mechanistic similarity. While coverage was low for this model, this was driven by the fact that this model does not force predictions when good analogs are unavailable. The analogs in this model are selected by first considering mechanistic similarity as opposed to the traditional approach of choosing surrogates based on structural similarity. While the model has low coverage and is undoubtedly conservative, it has relatively high accuracy and very high sensitivity, making it useful for screening purposes to eliminate chemicals within the domain of applicability that are legitimate sensitization concerns.

While the models offer moderate overall balanced accuracy, some of the models excel at identifying either true sensitizers or true non-sensitizers, as supported by high sensitivity and high specificity. Specifically, PredSkin, QSAR Toolbox, and CAESAR each had a sensitivity of 80% or greater for both data sets, with PredSkin almost achieving perfect sensitivity for both data sets (98% for Basketter et al. (2014) and 100% for HSDB). Specificity was less consistent, but TIMES-SS achieved 100% specificity, while Toxtree, REACHAcross^™^, and Derek achieved 80% or greater for the Basketter et al. (2014) data set. The Danish QSAR Database achieved 84% specificity for the HSDB data set, but none of the remaining models attained high specificity for the HSDB data set, likely reflecting that this was a more diverse data set and consisted of fewer chemicals likely to be in the model training sets. In some cases, high sensitivity may come at the expense of specificity and *vice versa*; however, this may be a tradeoff hazard assessors are willing to make, especially if they are using a combination of models. This approach may be more informative than the traditional approach that relies only on overall accuracy of a single model. Verheyen et al. (2017) have demonstrated that the combination of two QSAR models improves prediction accuracy compared to the use of an individual model, but using three or more models reduces the prediction accuracy.

Of note, our analysis did not include a comparison of each of the two test sets (i.e., Basketter et al., 2014 and HSDB) to the training sets of each of the 8 *in silico* models. This was because the training sets of several of the models are not publicly available; therefore, the overlap of each of the test sets with the training sets from each of the 8 *in silico* models could not be assessed. Depending on the degree of overlap, the global performance metrics could be skewed in favor of those models that have a high degree of overlap between the test and training sets. Nevertheless, we feel that this analysis would not significantly alter our assessment, as hazard assessors carrying out screening assessments do not typically compare their test set with the training set before running an analysis with an *in silico* tool. Instead, assessors consider whether the chemical falls within the applicability domain or the probability of accuracy to determine whether the prediction is reliable.

Finally, although many models offer advantages over others, a disadvantage common to all models is the availability of a universal, accurate, and complete structural input parameter. CASRNs aim to fulfill this role; however, the CASRNs are often incorrectly used interchangeably, and many chemicals have more than one CASRN. SMILES strings come with their own set of issues, for example, some of the SMILES strings sourced from ChemIDplus Advanced were provided in a format to reflect their multi-positional substituents. While this is the accurate SMILES string presentation for these chemicals, this unspecified or undefined format creates two major problems: first, many models simply cannot read this unspecified format, and second, other models force a prediction based on the unspecified SMILES string, which can sometimes produce inaccurate predictions. Additionally, various SMILES string formats (e.g., generic, isomeric, etc.) can produce different results in the same model for the same chemical. In order for *in silico* models to be truly reliable and incorporated into regulatory applications, these very basic issues need to be resolved.

## 5 Conclusions

Overall, most models performed with approximately 70–80% accuracy on both the Basketter et al. (2014) and HSDB data sets, which is comparable to the accuracy achieved by the LLNA model (74–82%) when compared to human skin sensitization results (Hoffman et al., 2018; Urbisch et al., 2015). Model performance indicates that *in silico* predictive models can offer a quick, convenient, and inexpensive tool to assess skin sensitization in humans, with the caveat that sensitivity and specificity remain far from perfect – although it should be noted that the predictivity of the LLNA test for humans has its own concerns (Anderson et al., 2011). The accuracy in terms of concordant classification between the guinea pig assay and the LLNA is 77%, and the reproducibility of the various skin sensitization assays (patch test, LLNA, Buehler, GPMT) ranges from approximately 75% to 95% (Hoffmann, 2015; Luechtefeld et al., 2016).

Some of the models discussed in this assessment have been developed further, increasing the amount of data used for the prediction, such as UL’s RASAR (Luechtefeld et al., 2018a). Additionally, although only binary predictions were used in this analysis, Derek also provides quantitative potency predictions for alerting chemicals (Canipa et al., 2017). More recently, Lhasa has released a DA (Macmillan and Chilton, 2019), which combines Derek predictions with *in chemico*/*in vitro* results as an alternative to animal testing. QSAR Toolbox has also released an update (Version 4.4), which includes a new automated workflow for skin sensitization as well as various other profiler and database updates.

Going forward, improving *in silico* skin sensitization predictions for humans will require, first and foremost, a concerted effort by the community to create and curate larger data sets with high-quality, transparent human skin sensitization data over a diverse set of chemicals. Additionally, our analysis pointed to several factors that can likely improve *in silico* tools: improved quality and consistency of human data in model training sets, as Alves et al. (2016a; 2018) demonstrated; transparent reporting of the probability of accuracy; and explicit modeling of metabolism. Further, none of the models considered addressed steric effects – likely due to the computational complexity of 3D approaches or lack of data on steric properties – therefore, it is possible that some of the inaccuracies in the models simply reflect the trade-off of reducing molecular complexity by using a 2D model. Lastly, it is likely that a better understanding of molecular mechanisms and building similarity metrics that reflect mechanism will improve model performance, as well as the incorporation of *in chemico* data that can provide quick read-outs of possible molecular mechanisms. This is especially true if models want to begin assessing potency instead of focusing on binary categorization (Luechtefeld et al., 2015). In conclusion, while it is clear that *in silico* models have much to offer hazard assessors for prioritization and screening level assessments, it is also true that there remains work to be done.

## Supplementary Material

Supplementary Material

## Figures and Tables

**Tab. 1:** Overview of the models

Model	Version used in this analysis; (publisher, year)	Molecular input used	Approach/Endpoint	Availability	Methodology	Results*
**PredSkin**	2.0 (LabMol, 2016)	SMILES	Human skin sensitization – binary prediction	Free	QSAR	Predicts whether chemical will be a skin sensitizer (Binary results for human prediction)
**Toxtree**	2.6.13 (Idea Consult, Ltd., 2015)	SMILES	Skin sensitization reactivity domains decision tree	Free	Structural alerts	Identifies structural alerts within target chemical
**QSAR Toolbox**	4.1 (OECD, 2017)	SMILES	Automated workflow for skin sensitization	Free	Read-across/skin metabolism simulation	Predicts whether chemical will be a skin sensitizer (Binary results*)
**Danish QSAR Database**	Not specified but updated in 2016 (NFI, 2016)	CASRN	Allergic contact dermatitis in guinea pig and human	Free	Battery algorithm based on three individual QSAR models	Predicts whether chemical will be a skin sensitizer (Binary results for human prediction)
**CAESAR (Skin sensitization)**	2.1.6 (IRCCS, 2017)	SMILES	Skin sensitization model	Free	QSAR	Predicts whether chemical will be a skin sensitizer (Binary results)
**REACHAcross^™^**	3.1.4 (UL, 2017)	CASRN	Skin sensitization endpoint	Commercial	QSAR/read-across	Predicts whether chemical will be a skin sensitizer (Binary results*)
**TIMES-SS**	2.28.1.6 (OASIS-LMC, 2017)	SMILES	Skin sensitization model	Commercial	QSAR/skin metabolism simulation	Predicts whether chemical will be a skin sensitizer (Binary results*)
**Derek Nexus**	6.0.1 (Lhasa Limited, 2018)	SMILES	Skin sensitization endpoint**	Commercial	Expert knowledge-based system	Predicts whether chemical will be a skin sensitizer based on structural alerts (Binary results*)

Summary of the administrative information, availability, and methodology of each *in silico* model as well as whether it predicts binary or potency outcomes.

IRCCS, Instituto di Ricerche Farmacologiche Mario Negri; LMC, Laboratory of Mathematical Chemistry; NFI, National Food Institute, Technical University of Denmark; UL, Underwriters Laboratories.

* Binary results were used in this analysis. TIMES-SS potency results were converted to binary results. QSAR Toolbox and Derek Nexus offer potency scales in addition to binary outputs; however, binary results were used for this analysis. REACHAcross^™^ offers predictions in terms of GHS Categories; however, binary results were used for this analysis.

** Chemicals were evaluated against the skin sensitization endpoint using mammal as the selected species. Perceive tautomers, perceive mixtures, and match alerts without rules options were unselected.

**Tab. 2:** Data set descriptors

Descriptor	n	%
Basketter et al., 2014 **data set**		
Total chemicals in final data set	131	100
Sensitizers	107	82
Human Class 1	15	11
Human Class 2	21	16
Human Class 3	24	18
Human Class 4	19	15
Human Class 5	28	21
Non-sensitizers (Human Class 6)	24	18
SMILES string unavailable*	8	6
CASRN issues	2	2
**HSDB data set**		
Total chemicals in final data set	375	100
Sensitizers	234	62
Non-sensitizers	141	38
SMILES string unavailable*	14	4
CASRN issues	0	0

Summary of the distribution of sensitizers across sensitization categories for both the Basketter et al. (2014) and HSDB data sets. For the Basketter et al. (2014) data set, the chemical distributions are shown in both categorical and binary distributions, while the HSDB data set distribution is simply binary. If a SMILES string was unavailable, this indicates that it was not available in ChemIDplus Advanced. A CASRN issue indicates that a chemical may be represented by more than one CASRN; in this case, a representative CASRN was used.

Source: Basketter et al. (2014);

* In ChemIDplus Advanced

**Tab. 3:** Concordance of the skin sensitization status for both the Basketter et al. (2014) and HSDB data sets compared to the harmonized CLP skin sensitization classification statuses

Data source	Chemicals with harmonized classifications in CLP n (%)	Concordance (%)	Concordant positive n (%)	Concordant negative n (%)	Discordant positive n (%)	Discordant negative n (%)
Basketter et al., 2014	54 (41%)	78	32 (100%)	10 (45%)	12 (55%)	0 (0%)
**HSDB**	169 (45%)	64	72 (92%)	37 (41%)	54 (59%)	6 (8%)

To assess the concordance of the skin sensitization statuses in each data set with the more standardized approach adopted in the CLP, we compared each data set with the harmonized skin sensitization classifications as assigned according to the CLP (ECHA, 2018a).

**Tab. 4:** Comparison of the concordant sensitization statuses for the HSDB data set to the Basketter et al. (2014) data set for chemicals present in both data sets

Sensitization status	n	%
**Binary**		
**Sensitizer**	31	78
**Non-sensitizer**	1	100
**Total**	32	78
**Potency categories**		
**Human Category 1**	5	100
**Human Category 2**	9	90
**Human Category 3**	6	86
**Human Category 4**	7	88
**Human Category 5**	4	40
**Human Category 6**	1	100

Concordance of the predictions for chemicals that are in both data sets (n = 41). For the potency comparisons, the Basketter et al. (2014) categorizations were used for both data sets when evaluating concordance by potency category.

**Tab. 5:** Summary of optimized settings by model

Model	Optimized settings	Prediction format
**PredSkin**	Within applicability domain; probability of accuracy > 70%	Binary
**Toxtree**	None*	Binary
**QSAR Toolbox**	Within applicability domain; metabolism incorporated	Binary (in automated workflow)
**REACHAcross^™^**	Within applicability domain; probability of accuracy > 70%	Binary and GHS Category format; binary used for this analysis
**Danish QSAR Database**	Within applicability domain	Binary
**CAESAR**	Within applicability domain	Binary
**TIMES-SS**	Within applicability domain; metabolism incorporated	Potency; converted to binary for this analysis
**Derek**	All predictions with the exception of non-sensitizer with misclassified and/or unclassified	Likelihood level; converted to binary for this analysis

For each model, optimized settings were applied to obtain a high confidence result. Additionally, most models provided predictions in binary form; however, some models provided predictions in potency format, and those predictions were converted to binary format.

* Toxtree is a structural alert model and, therefore, does not have any optimization features. Consequently, the model output was considered to be the high confidence result without any further optimization.

**Tab. 6:** Evaluation of Category 5 skin sensitization status on model accuracy and balanced accuracy

Model	Accuracy		Balanced accuracy	
	Category 5 positive	Category 5 negative	Category 5 positive	Category 5 negative
**PredSkin**	87	75	55	56
**Toxtree**	69	73	73	72
**QSAR Toolbox**	84	74	68	66
**REACHAcross^™^**	79	85	83	83
**Danish QSAR Database**	61	71	66	71
**CAESAR**	83	79	83	75
**TIMES-SS**	78	89	88	88
**Derek**	86	86	86	83

The effect of model accuracies and balanced accuracies for high confidence results when the skin sensitization status of the Category 5 chemicals from the Basketter et al. (2014) data set are changed from positive to negative.

## Footnotes

1 doi:10.14573/altex.1911261s

2 ChemIDplus Advanced. https://chem.nlm.nih.gov/chemidplus/ (accessed 5 October 2017)

3 Hazardous Substances Data Bank. https://toxnet.nlm.nih.gov/newtoxnet/hsdb.htm (accessed 5 October 2017)

## References

[R1] Akhtar A (2015). The flaws and human harms of animal experimentation. Camb Q Healthc Ethics 24, 407–419. doi:10.1017/s0963180115000079 26364776 PMC4594046

[R2] Alves VM, Capuzzi SJ, Muratov E (2016a). QSAR models of human data can enrich or replace LLNA testing for human skin sensitization. Green Chem 18, 6501–6515. doi:10.1039/c6gc01836j 28630595 PMC5473635

[R3] Alves V, Muratov E, Capuzzi S (2016b). Alarms about structural alerts. Green Chem 18, 4348–4360. doi:10.1039/c6gc01492e 28503093 PMC5423727

[R4] Alves VM, Capuzzi SJ, Braga RC (2018). A perspective and a new integrated computational strategy for skin sensitization assessment. ACS Sustainable Chem Eng 6, 2845–2859. doi:10.1021/acssuschemeng.7b04220

[R5] Anderson SE, Siegel PD and Meade BJ (2011). The LLNA: A brief review of recent advances and limitations. J Allergy (Cairo) 2011, 424203. doi:10.1155/2011/424203 21747867 PMC3124934

[R6] Bailey J, Thew M and Balls M (2014). An analysis of the use of animal models in predicting human toxicology and drug safety. Altern Lab Anim 42, 181–199. doi:10.1177/026119291404200306 25068930

[R7] Bailey J, Thew M and Balls M (2015). Predicting human drug toxicity and safety via animal tests: Can any one species predict drug toxicity in any other, and do monkeys help? Altern Lab Anim 43, 393–403. doi:10.1177/026119291504300607 26753942

[R8] Basketter DA (2009). The human repeated insult patch test in the 21^st^ century: A commentary. Cutan Ocul Toxicol 28, 49–53. doi:10.1080/15569520902938032 19514927

[R9] Basketter DA, Clewell H, Kimber I (2012). A road-map for the development of alternative (non-animal) methods for systemic toxicity testing. ALTEX 29, 3–91. doi:10.14573/altex.2012.1.003 22307314

[R10] Basketter DA, Alépée N, Ashikaga T (2014). Categorization of chemicals according to their relative human skin sensitizing potency. Dermatitis 25, 11–21. doi:10.1097/der.0000000000000003 24407057

[R11] Bauch C, Kolle SN, Ramirez T (2012). Putting the parts together: Combining in vitro methods to test for skin sensitizing potentials. Regul Toxicol Pharmacol 63, 489–504. doi:10.1016/j.yrtph.2012.08.014 22659254

[R12] Canipa SJ, Chilton ML, Hemingway R (2017). A quantitative in silico model for predicting skin sensitization using a nearest neighbours approach within expert-derived structure-activity alert spaces. J Appl Toxicol 37, 985–995. doi:10.1002/jat.3448 28244128

[R13] Cashman MW, Reutemann PA and Ehrlich A (2012). Contact dermatitis in the united states: Epidemiology, economic impact, and workplace prevention. Dermatol Clin 30, 87–98. doi:10.1016/j.det.2011.08.004 22117870

[R14] Chaudhry Q, Piclin N, Cotterill J (2010). Global QSAR models of skin sensitisers for regulatory purposes. Chem Cent J 4, Suppl 1, S5. doi:10.1186/1752-153x-4-s1-s5 20678184 PMC2913332

[R15] Cronin MTD (2010). Quantitative structure-activity relationships (QSARs) – Applications and methodology. In Puzyn T, Leszczynski J and Cronin MT (eds.), Recent Advances in QSAR Studies: Methods and Applications (3–11). Dordrecht, The Netherlands: Springer. doi:10.1007/978-1-4020-9783-6_1

[R16] Cronin MTD and Madden JC (2010). In silico toxicology – An introduction. In Cronin MTD and Madden JC (eds.), In Silico Toxicology (1–10). Cambridge, UK: RSC Publishing. doi:10.1039/9781849732093

[R17] Dreisbach RH (ed.) (1977). Handbook of Poisoning: Diagnosis & Treatment. Lange Medical Publications.

[R18] EC – European Commission (2016). Ban on Animal Testing – Internal Market, Industry, Entrepreneurship and SMEs. https://ec.europa.eu/growth/sectors/cosmetics/animal-testing_en (accessed 30.04.2018).

[R19] ECHA – European Chemicals Agency (2018a). Table of harmonised entries in Annex VI to CLP. https://echa.europa.eu/information-on-chemicals/annex-vi-to-clp

[R20] ECHA (2018b). Harmonised classification and labelling (CLH). https://echa.europa.eu/regulations/clp/harmonised-classification-and-labelling

[R21] Enoch SJ, Ellison CM, Schultz TW (2011). A review of the electrophilic reaction chemistry involved in covalent protein binding relevant to toxicity. Crit Rev Toxicol 41, 783–802. doi:10.3109/10408444.2011.598141 21809939

[R22] EU – European Union (2009). Regulation (EC) No 1223/2009 of the European Parliament and of the Council of 30 November 2009 on cosmetic products. https://eur-lex.europa.eu/legal-content/EN/ALL/?uri=CELEX%3A32009R1223

[R23] Fitzpatrick JM, Roberts DW and Patlewicz G (2016). What determines skin sensitization potency: Myths, maybes and realities. The 500 molecular weight cut-off: An updated analysis. J Appl Toxicol 37, 105–116. doi:10.1002/jat.3348 27283458

[R24] Fitzpatrick JM, Roberts DW and Patlewicz G (2018). An evaluation of selected (Q)SARs/expert systems for predicting skin sensitisation potential. SAR QSAR Environ Res 29, 439–468. doi:10.1080/1062936x.2018.1455223 29676182 PMC6077848

[R25] Gerberick GF, Ryan CA, Kern PS (2005). Compilation of historical local lymph node data for evaluation of skin sensitization alternative methods. Dermatitis 16, 157–202. doi:10.1097/01206501-200512000-00002 16536334

[R26] Hartung T (2008). Food for thought… On animal tests. ALTEX 25, 3–16. doi:10.14573/altex.2008.1.3 18360722

[R27] Hartung T (2009). Toxicology for the twenty-first century. Nature 460, 208–212. doi:10.1038/460208a 19587762

[R28] Hartung T, Luechtefeld T, Maertens A (2013). Integrated testing strategies for safety assessments. ALTEX 30, 3–18. doi:10.14573/altex.2013.1.003 23338803 PMC3800026

[R29] Hartung T (2016). Making big sense from big data in toxicology by read-across. ALTEX 33, 83–93. doi:10.14573/altex.1603091 27032088

[R30] Hayes WJ and Laws ER (eds.) (1991). Handbook of Pesticide Toxicology: Classes of pesticides. New York, NY, USA: Academic Press.

[R31] Hoffmann S (2015). LLNA variability: An essential ingredient for a comprehensive assessment of non-animal skin sensitization test methods and strategies. ALTEX 32, 379–383. doi:10.14573/altex.1505051 26168096

[R32] Hoffmann S, Kleinstreuer N, Alepee N (2018). Non-animal methods to predict skin sensitization (I): The Cosmetics Europe database. Crit Rev Toxicol 48, 344–358. doi:10.1080/10408444.2018.1429385 29474128

[R33] Jaworska J (2016). Integrated testing strategies for skin sensitization hazard and potency assessment – State of the art and challenges. Cosmet Toiletries 3, 16. doi:10.3390/cosmetics3020016

[R34] Johansson H and Lindstedt M (2014). Prediction of skin sensitizers using alternative methods to animal experimentation. Basic Clin Pharmacol Toxicol 115, 110–117. doi:10.1111/bcpt.12199 24548737

[R35] Kadivar S and Belsito DV (2015). Occupational dermatitis in health care workers evaluated for suspected allergic contact dermatitis. Dermatitis 26, 177–183. doi:10.1097/der.0000000000000124 26172487

[R36] Kimber I, Basketter DA, Gerberick GF (2002). Allergic contact dermatitis. Int Immunopharmacol 2, 201–211. doi:10.1016/s1567-5769(01)00173-4 11811925

[R37] Kleinstreuer NC, Hoffmann S, Alépée N (2018). Non-animal methods to predict skin sensitization (II): An assessment of defined approaches*. Crit Rev Toxicol 48, 359–374. doi:10.1080/10408444.2018.1429386 29474122 PMC7393691

[R38] Kostal J and Voutchkova-Kostal A (2016). CADRE-SS, an in silico tool for predicting skin sensitization potential based on modeling of molecular interactions. Chem Res Toxicol 29, 58–64. doi:10.1021/acs.chemrestox.5b00392 26650775

[R39] Leist M and Hartung T (2013). Inflammatory findings on species extrapolations: Humans are definitely no 70-kg mice. Arch Toxicol 87, 563–567. doi:10.1007/s00204-013-1038-0 23503654 PMC3604596

[R40] Luechtefeld T, Maertens A, McKim JM (2015). Pro,-babilistic hazard assessment for skin sensitization potency by dose-response modeling using feature elimination instead of quantitative structure-activity relationships. J Appl Toxicol 35, 1361–1371. doi:10.1002/jat.3172 26046447 PMC4805435

[R41] Luechtefeld T, Maertens A, Russo DP (2016). Analysis of publically available skin sensitization data from REACH registrations 2008–2014. ALTEX 33, 135–148. doi:10.14573/altex.1510055 26863411 PMC5546098

[R42] Luechtefeld T and Hartung T (2017). Computational approaches to chemical hazard assessment. ALTEX 34, 459–478. doi:10.14573/altex.1710141 29101769 PMC5848496

[R43] Luechtefeld T, Marsh D, Rowlands C (2018a). Machine learning of toxicological big data enables read-across structure activity relationships (RASAR) outperforming animal test reproducibility. Toxicol Sci 165, 198–212. doi:10.1093/toxsci/kfy152 30007363 PMC6135638

[R44] Luechtefeld T, Rowlands C and Hartung T (2018b). Big-data and machine learning to revamp computational toxicology and its use in risk assessment. Toxicol Res 7, 732–744. doi:10.1039/c8tx00051d PMC6116175 30310652

[R45] Macmillan DS and Chilton ML (2019). A defined approach for predicting skin sensitisation hazard and potency based on the guided integration of in silico, in chemico and in vitro data using exclusion criteria. Regul Toxicol Pharmacol 101, 35–47. doi:10.1016/j.yrtph.2018.11.001 30439387

[R46] Maertens A, Anastas N, Spencer PJ (2014). Food for thought… Green toxicology. ALTEX 31, 243–249. doi:10.14573/altex.1406181 25061898

[R47] Maertens A and Hartung T (2018). Green toxicology-know early about and avoid toxic product liabilities. Toxicol Sci 161, 285–289. doi:10.1093/toxsci/kfx243 29267930

[R48] NRC – National Research Council, Division on Earth and Life Studies, Institute for Laboratory Animal Research, Board on Environmental Studies and Toxicology, Committee on Toxicity Testing and Assessment of Environmental Agents (2007). Toxicity Testing in the 21^st^ Century: A Vision and a Strategy. National Academies Press. doi:10.17226/25135

[R49] OECD – Organisation for Economic Co-operation and Development (2012). The Adverse Outcome Pathway for Skin Sensitisation Initiated by Covalent Binding to Proteins. Part 1: Scientific Evidence. OECD Series on Testing and Assessment, No. 168 OECD Publishing, Paris. doi:10.1787/9789264221444-en

[R50] OECD (2016). Guidance Document on the Reporting of Defined Approaches and Individual Information Sources to be Used within Integrated Approaches to Testing and Assessment (IATA) for Skin Sensitisation. OECD Series on Testing and Assessment, No. 256 OECD Publishing, Paris. doi:10.1787/9789264279285-en

[R51] OECD (2018a). Test No. 442D: In Vitro Skin Sensitisation ARE-Nrf2 Luciferase Test Method: ARE-Nrf2 Luciferase Test Method. OECD Guidelines for the Testing of Chemicals, Section 4. OECD Publishing, Paris. Adopted 25 June 2018. doi:10.1787/9789264229822-en

[R52] OECD (2018b). Test No. 442E: In Vitro Skin Sensitisation: In Vitro Skin Sensitisation assays addressing the Key Event on activation of dendritic cells on the Adverse Outcome Pathway for Skin Sensitisation. OECD Guidelines for the Testing of Chemicals, Section 4. OECD Publishing, Paris. doi:10.1787/9789264264359-en.

[R53] OECD (2019). Test No. 442C: In Chemico Skin Sensitisation. Assays addressing the Adverse Outcome Pathway key event on covalent binding to proteins. OECD Guidelines for the Testing of Chemicals, Section 4. OECD Publishing, Paris. doi:10.1787/9789264229709-en

[R54] Pohanish RP (ed.) (2012). Sittig’s Handbook of Toxic and Hazardous Chemicals and Carcinogens (Sixth Edition). Oxford, UK: William Andrew Publishing. doi:10.1016/b978-1-4377-7869-4.00010-2

[R55] Pound P and Bracken MB (2014). Is animal research sufficiently evidence based to be a cornerstone of biomedical research? BMJ 348, g3387. doi:10.1136/bmj.g3387 24879816

[R56] Raunio H (2011). In silico toxicology – Non-testing methods. Front Pharmacol 2, 33. doi:10.3389/fphar.2011.00033 21772821 PMC3129017

[R57] Roberts DW and Patlewicz G (2009). Chemistry based non-animal predictive modeling for skin sensitization. In Devillers J (ed.), Ecotoxicology Modeling (61–83). Boston, MA, USA: Springer. doi:10.1007/978-1-4419-0197-2_3

[R58] Sailstad DM, Hattan D, Hill RN (2001). ICCVAM evaluation of the murine local lymph node assay. Reg Toxicol Pharmacol 34, 249–257. doi:10.1006/rtph.2001.1496 11754529

[R59] Schmidt M, Raghavan B, Muller V (2010). Crucial role for human toll-like receptor 4 in the development of contact allergy to nickel. Nat Immunol 11, 814–819. doi:10.1038/ni.1919 20711192

[R60] Smith Pease CK, Basketter DA and Patlewicz GY (2003). Contact allergy: The role of skin chemistry and metabolism. Clin Exp Dermatol 28, 177–183. doi:10.1046/j.1365-2230.2003.01239.x 12653709

[R61] Teubner W, Mehling A, Schuster PX (2013). Computer models versus reality: How well do in silico models currently predict the sensitization potential of a substance. Regul Toxicol Pharmacol 67, 468–485. doi:10.1016/j.yrtph.2013.09.007 24090701

[R62] Thyssen JP, Linneberg A, Menne T (2007). The epidemiology of contact allergy in the general population – Prevalence and main findings. Contact Dermatitis 57, 287–299. doi:10.1111/j.1600-0536.2007.01220.x 17937743

[R63] UN – United Nations. (2017). Globally Harmonized System of Classification and Labelling of Chemicals (GHS) (Rev.7) doi:10.18356/e18d11a0-en

[R64] Urbisch D, Mehling A, Guth K (2015). Assessing skin sensitization hazard in mice and men using non-animal test methods. Regul Toxicol Pharmacol 71, 337–351. doi:10.1016/j.yrtph.2014.12.008 25541156

[R65] Verheyen GR, Braeken E, Van Deun K (2017). Evaluation of in silico tools to predict the skin sensitization potential of chemicals. SAR QSAR Environ Res 28, 59–73. doi:10.1080/1062936x.2017.1278617 28105856

[R66] Warshaw EM, Hagen SL, Sasseville D (2017). Occupational contact dermatitis in mechanics and repairers referred for patch testing: Retrospective analysis from the North American contact dermatitis group 1998–2014. Dermatitis 28, 47–57. doi:10.1097/der.0000000000000231 27775971

[R67] Wilm A, Kuhnl J and Kirchmair J (2018). Computational approaches for skin sensitization prediction. Crit Rev Toxicol 48, 738–760. doi:10.1080/10408444.2018.1528207 30488745

