# Supplementary material for: Evaluation of the Global Performance of Eight In Silico Skin Sensitization Models Using Human Data
Source: ALTEX. Author manuscript; Available in PMC 2024 Aug 10. (PMC11316520; doi:10.14573/altex.1911261)
Supplement: Supplementary Material [file NIHMS2014200-supplement-Supplementary_Material.pdf]

Golden et al.:

Evaluation of the Global Performance of Eight *In Silico* Skin Sensitization Models Using Human Data

Supplementary Data

Tab. S1: Basketter et al. (2014) data set

Number	CAS number	Chemical name	SMILES string	Potency category	Binary sensitization status
1	526-37-4	Atranol	Cc1cc(c(c(c1)O)C=O)O	Human Class 1	Sensitizer
2	7787-56-6	Beryllium sulfate	S(=O)(=O)([O-])[O-].O.O.O.O.[Be+2]	Human Class 1	Sensitizer
3	57074-21-2	Chloroatranol	n/a	Human Class 1	Sensitizer
4	624-49-7	Dimethyl fumarate	C(\C=C\C(OC)=O)(OC)=O	Human Class 1	Sensitizer
5	97-00-7	Dinitrochlorobenzene	c1(cc(ccc1Cl)[N+](=O)[O-])[N+](=O)[O-]	Human Class 1	Sensitizer
6	886-38-4	Diphenylcyclopropenone	C1(C(c2ccccc2)=C1c1ccccc1)=O	Human Class 1	Sensitizer
7	302-01-2	Hydrazine	NN	Human Class 1	Sensitizer
8*	55965-84-9/ 96118-96-6	MCI/MI	c1(Cl)cc(=O)n(s1)C.c1(n(scc1)C)=O	Human Class 1	Sensitizer
9	7487-94-7	Mercuric chloride	[Hg](Cl)Cl	Human Class 1	Sensitizer
10	2682-20-4	MI	Cn1c(=O)ccs1	Human Class 1	Sensitizer
11	106-50-3	p-Phenylenedimaine	c1cc(ccc1N)N	Human Class 1	Sensitizer
12	492-89-7	Pentadecylcatechol	CCCCCCCCCCCCCCCc1c(O)c(O)ccc1	Human Class 1	Sensitizer
13	7778-50-9	Potassium dichromate	[Cr](O[Cr](=O)(=O)[O-])(=O)(=O)[O-].[K+].[K+]	Human Class 1	Sensitizer
14	2892-62-8	Squaric acid dibutylester	C1(=O)C(OCCCC)=C(OCCCC)C1=O	Human Class 1	Sensitizer
15	1154-59-2	Tetrachlorosalicylanilide	c1(C(Nc2cc(c(Cl)cc2)Cl)=O)c(c(cc(c1)Cl)Cl)O	Human Class 1	Sensitizer
16	95-55-6	2-Aminophenol	c1(c(cccc1)O)N	Human Class 2	Sensitizer
17	2634-33-5	Benzisothiazolinone	c1ccc2c(c1)c(=O)[nH]s2	Human Class 2	Sensitizer
18	104-55-2	Cinnamal	c1(ccccc1)/C=C/C=O	Human Class 2	Sensitizer
19	10124-43-3	Cobalt sulfate	S(=O)(=O)([O-])[O-].[Co+2]	Human Class 2	Sensitizer
20	141-05-9	Diethyl maleate	C(=C/C(=O)OCC)\C(OCC)=O	Human Class 2	Sensitizer
21	109-55-7	Dimethylaminopropylamine	C(N(C)C)CCN	Human Class 2	Sensitizer
22	50-00-0	Formaldehyde	C=O	Human Class 2	Sensitizer
23	111-30-8	Glutaraldehyde	C(CC=O)CC=O	Human Class 2	Sensitizer
24	107-22-2	Glyoxal	C(=O)C=O	Human Class 2	Sensitizer
25	13453-07-1	Gold chloride	[Au](Cl)(Cl)Cl	Human Class 2	Sensitizer
26	31906-04-4	Hydroxyisohexyl-3-cyclohexene carboxaldehyde	CC(C)(CCCC1=CCC(CC1)C=O)O	Human Class 2	Sensitizer
27	97-54-1	Isoeugenol	C/C=C/c1ccc(c(c1)OC)O	Human Class 2	Sensitizer
28	1166-52-5	Lauryl gallate	c1(cc(c(O)c(c1)O)O)C(OCCCCCCCCCCCC)=O	Human Class 2	Sensitizer
29	111-12-6	Methyl heptine carbonate	C(#CC(OC)=O)CCCCC	Human Class 2	Sensitizer
30	35691-65-7	Methyldibromo glutaronitrile	C(CCC#N)(C#N)(CBr)Br	Human Class 2	Sensitizer
31	7440-02-0	Nickel	[Ni]	Human Class 2	Sensitizer
32	5307-14-2	Nitro-4-phenylenediamine	c1(c(ccc(c1)N)N)[N+](=O)[O-]	Human Class 2	Sensitizer
33	8007-00-9	Peru balsam	n/a	Human Class 2	Sensitizer
34	121-79-9	Propyl gallate	c1(cc(c(c(c1)O)O)O)C(=O)OCCC	Human Class 2	Sensitizer
35	4080-31-3	Quaternium-15	[N+]12(CN3CN(C1)CN(C2)C3)C\C=C\Cl.[ClH-]	Human Class 2	Sensitizer

doi:10.14573/altex.1911261s

Number	CAS number	Chemical name	SMILES string	Potency category	Binary sensitization status
36	54-64-8	Thimerosal	c1(c(cccc1)C(=O)[O-])S[Hg]CC.[Na+]	Human Class 2	Sensitizer
37	96-27-5	Thioglycerol	C([C@@H](CS)O)O	Human Class 2	Sensitizer
38	615-50-9	Toluene diamine sulfate	Cc1cc(ccc1N)N.OS(=O)(=O)O	Human Class 2	Sensitizer
39	514-10-3	Abietic acid (colophony)	C1[C@@H]2[C@@]([C@@H]3C(C=C(C(C)C)CC3)=C1)(CCC[C@]2(C(O)=O)C)C	Human Class 3	Sensitizer
40	1405-87-4	Bacitracin	CC[C@H](C)[C@H](N)C1=N[C@@H](CS1)C(=O)N[C@@H](CC(C)C)C(=O)N[C@H](CCC(=O)O)C(=O)N[C@@H]([C@@H](C)CC)C(=O)N[C@H]2CCCCNC(=O)[C@H](CC(=O)N)NC(=O)[C@@H](CC(=O)O)NC(=O)[C@H](Cc3c[nH]cn3)NC(=O)[C@@H](Cc4ccccc4)NC(=O)[C@@H](NC(=O)[C@@H](CCCN)NC2=O)[C@@H](C)CC	Human Class 3	Sensitizer
41	94-36-0	Benzoyl peroxide	c1(C(OOC(c2ccccc2)=O)=O)ccccc1	Human Class 3	Sensitizer
42	1675-54-3	Bisphenol A glycidyl ether	C(c1ccc(cc1)OCC1CO1)(c1ccc(cc1)OCC1CO1)(C)C	Human Class 3	Sensitizer
43	2426-08-6	Butylglycidylether	C1[C@@H](O1)COCCCC	Human Class 3	Sensitizer
44	50-53-3	Chlorpromazine	CN(C)CCCN1c2ccccc2Sc3c1cc(cc3)Cl	Human Class 3	Sensitizer
45	104-54-1	Cinnamic alcohol	c1(ccccc1)/C=C/CO	Human Class 3	Sensitizer
46	5392-40-5	Citral	C(=C\C=O)(/CC/C=C(/C)C)C	Human Class 3	Sensitizer
47	91-64-5	Coumarin	c12c(ccc(o1)=O)cccc2	Human Class 3	Sensitizer
48	107-15-3	Ethylenediamine	C(CN)N	Human Class 3	Sensitizer
49	97-53-0	Eugenol	COc1cc(ccc1O)CC=C	Human Class 3	Sensitizer
50	4602-84-0	Farnesol	C(=C\CC\C(=C\CO)C)(\CC\C=C(\C)C)C	Human Class 3	Sensitizer
51	30618-84-9	Glycerylmonothioglycolate	C(C(COC(=O)CS)O)O	Human Class 3	Sensitizer
52	123-31-9	Hydroquinone	c1(ccc(cc1)O)O	Human Class 3	Sensitizer
53	818-61-1	Hydroxyethyl acrylate	C(OCCO)(C=C)=O	Human Class 3	Sensitizer
54	39236-46-9	Imidazolidinyl urea	N1([C@@H](C(NC1=O)=O)NC(NCNC(N[C@@H]1N(C(=O)NC1=O)CO)=O)=O)CO	Human Class 3	Sensitizer
55	149-30-4	Mercaptobenzothiazole	c1ccc2c(c1)nc(s2)S	Human Class 3	Sensitizer
56	13706-86-0	Methyl-2,3-hexanedione	CC(C)CC(=O)C(=O)C	Human Class 3	Sensitizer
57	55-55-0	Metol	CNc1ccc(cc1)O.CNc1ccc(cc1)O.OS(=O)(=O)O	Human Class 3	Sensitizer
58	61-33-6	Penicillin	CC1([C@@H](N2[C@H](S1)[C@@H](C2=O)NC(=O)Cc3ccccc3)C(=O)O)C	Human Class 3	Sensitizer
59	93-99-2	Phenyl benzoate	c1(C(Oc2ccccc2)=O)ccccc1	Human Class 3	Sensitizer
60	137-26-8	Tetramethylthiuram disulfide	CN(C)C(=S)SSC(=S)N(C)C	Human Class 3	Sensitizer
61	122-40-7	Amylcinnamal	c1(\C=C(\CCCCC)C=O)ccccc1	Human Class 4	Sensitizer
62	101-85-9	Amylcinnamal alcohol	c1(\C=C(/CCCCC)CO)ccccc1	Human Class 4	Sensitizer
63	62-53-3	Aniline	c1ccc(cc1)N	Human Class 4	Sensitizer
64	94-09-7	Benzocaine	c1cc(C(OCC)=O)ccc1N	Human Class 4	Sensitizer
65	52-51-7	Bronopol	C(C(CO)([N+](=O)[O-])Br)O	Human Class 4	Sensitizer
66	99-49-0	Carvone	CC(=C)C1CC=C(C)C(=O)C1	Human Class 4	Sensitizer
67	55-56-1	Chlorhexidine gluconate	c1(NC(NC(NCCCCCCNC(NC(Nc2ccc(Cl)cc2)=N)=N)=N)=N)ccc(Cl)cc1	Human Class 4	Sensitizer
68	9003-32-1	Ethyl acrylate	CCOC(C=C)=O	Human Class 4	Sensitizer
69	97-90-5	Ethyleneglycol dimethacrylate	C(OCCOC(C(C)=C)=O)(C(C)=C)=O	Human Class 4	Sensitizer
70	106-24-1	Geraniol	C(\CC\C=C(\C)C)(=C\CO)C	Human Class 4	Sensitizer
71	6259-76-3	Hexyl salicylate	c1(c(cccc1)O)C(OCCCCCC)=O	Human Class 4	Sensitizer
72	107-75-5	Hydroxcitronellal	CC(CCCC(C)(C)O)CC=O	Human Class 4	Sensitizer
73	55406-53-6	Iodopropynyl butyl carbamate	N(C(OCC#CI)=O)CCCC	Human Class 4	Sensitizer
74	8063-07-8	Kanamycin	n/a	Human Class 4	Sensitizer

Number	CAS number	Chemical name	SMILES string	Potency category	Binary sensitization status
75	80-54-6	p-tert-butyl-a-methylhydrocinnamal	CC(Cc1ccc(cc1)C(C)(C)C)C=O	Human Class 4	Sensitizer
76	78-70-6	Linalool	C([C@@](C=C)(C)O)C\C=C(\C)C	Human Class 4	Sensitizer
77	80-62-6	Methylmethacrylate	C(C(C)=C)(OC)=O	Human Class 4	Sensitizer
78	1404-04-2	Neomycin sulfate	O1[C@H](O[C@H]2[C@H](O[C@H]3[C@@H]([C@@H]([C@H](O3)CO)O[C@@H]3[C@@H]([C@H]([C@@H]([C@@H](O3)CN)O)O)N)O)[C@H]([C@@H](C[C@@H]2N)N)O)[C@@H]([C@H]([C@@H](O)[C@H]1CN)O)N	Human Class 4	Sensitizer
79	108-46-3	Resorcinol	c1c(cccc1O)O	Human Class 4	Sensitizer
80	104-46-1	Anethole	c1(ccc(cc1)OC)/C=C/C	Human Class 5	Sensitizer
81	150-13-0	4-Aminobenzoic acid	c1cc(ccc1C(=O)O)N	Human Class 5	Sensitizer
82	105-13-5	Anisyl alcohol	c1(ccc(OC)cc1)CO	Human Class 5	Sensitizer
83	100-52-7	Benzaldehyde	c1ccc(cc1)C=O	Human Class 5	Sensitizer
84	8001-54-5	Benzalkonium chloride	c1(C[N+](C)(C)*)ccccc1.[ClH-]	Human Class 5	Sensitizer
85	120-51-4	Benzyl benzoate	c1(C(=O)OCc2ccccc2)ccccc1	Human Class 5	Sensitizer
86	118-58-1	Benzyl salicylate	c1(C(OCc2ccccc2)=O)c(cccc1)O	Human Class 5	Sensitizer
87	128-37-0	Butylated hydroxytoluene	c1(c(O)c(C(C)(C)C)cc(c1)C)C(C)(C)C	Human Class 5	Sensitizer
88	107-88-0	Butylene glycol	C([C@@H](C)O)CO	Human Class 5	Sensitizer
89	57-09-0	Cetrimide	C(CCCCCCCCCCCCCC)CC[N+](C)(C)C.[BrH-]	Human Class 5	Sensitizer
90	773-52-6	Cetylpyridinium chloride	n/a	Human Class 5	Sensitizer
91	106-22-9	Citronellol	C(CC/C=C(/C)C)(CCO)C	Human Class 5	Sensitizer
92	111-42-2	Diethanolamine	N(CCO)CCO	Human Class 5	Sensitizer
93	101-86-0	Hexylcinnamal	CCCCCC/C(=C/c1ccccc1)/C=O	Human Class 5	Sensitizer
94	50-23-7	Hydrocortisone	C[C@]12CCC(=O)C=C1CC[C@@H]3[C@@H]2[C@H](C[C@]4([C@H]3CC[C@@]4(C(=O)CO)O)C)O	Human Class 5	Sensitizer
95	67-63-0	Isopropanol	C(C)(C)O	Human Class 5	Sensitizer
96	110-27-0	Isopropyl myristate	C(CCCCCCCCCCCC)C(=O)OC(C)C	Human Class 5	Sensitizer
97**	138-86-3/ 5989-27-5	Limonene (not oxidized)	C([C@@H]1CCC(C)=CC1)(C)=C	Human Class 5	Sensitizer
98	119-36-8	Methyl salicylate	c1(c(cccc1)O)C(OC)=O	Human Class 5	Sensitizer
99	87-86-5	Pentachlorophenol	c1(c(c(c(Cl)c(c1Cl)Cl)Cl)Cl)O	Human Class 5	Sensitizer
100	122-99-6	Phenoxyethanol	c1(ccccc1)OCCO	Human Class 5	Sensitizer
101	94-13-3	Propyl paraben	c1c(O)ccc(C(OCCC)=O)c1	Human Class 5	Sensitizer
102	57-55-6	Propylene glycol	C([C@@H](C)O)O	Human Class 5	Sensitizer
103	110-86-1	Pyridine	c1ccncc1	Human Class 5	Sensitizer
104	110-44-1	Sorbic acid	C(=C\C=C\C)\C(O)=O	Human Class 5	Sensitizer
105	3380-34-5	Triclosan	c1(Oc2c(cc(Cl)cc2)Cl)c(cc(Cl)cc1)O	Human Class 5	Sensitizer
106	102-71-6	Triethanolamine	N(CCO)(CCO)CCO	Human Class 5	Sensitizer
107	121-33-5	Vanillin	c1(cc(ccc1O)C=O)OC	Human Class 5	Sensitizer
108	7446-70-0	Aluminum chloride	[Al](Cl)(Cl)Cl	Human Class 6	Non-sensitizer
109	50-81-7	Ascorbic acid	[C@H]1(C(=C(C(=O)O1)O)O)[C@H](CO)O	Human Class 6	Non-sensitizer
110	71-43-2	Benzene	c1ccccc1	Human Class 6	Non-sensitizer
111	71-36-3	Butanol	C(CC)CO	Human Class 6	Non-sensitizer
112	9004-54-0	Dextran	n/a	Human Class 6	Non-sensitizer
113	84-66-2	Diethyl phthalate	CCOC(=O)c1ccccc1C(=O)OCC	Human Class 6	Non-sensitizer
114	94271-03-1	Diethyl toluamide	c1(C(=O)N(CC)CC)cc(ccc1)C	Human Class 6	Non-sensitizer
115	67-68-5	Dimethylsulfoxide	CS(=O)C	Human Class 6	Non-sensitizer
116	50-99-7	Glucose	OC[C@H]([C@H]([C@@H]([C@H](C=O)O)O)O)O	Human Class 6	Non-sensitizer
117	56-81-5	Glycerol	C(CO)(CO)O	Human Class 6	Non-sensitizer
118	70-30-4	Hexachlorophene	c1(Cc2c(c(cc(c2Cl)Cl)Cl)O)c(c(cc(c1Cl)Cl)Cl)O	Human Class 6	Non-sensitizer
119	110-54-3	Hexane	C(CCC)CC	Human Class 6	Non-sensitizer
120	50-21-5	Lactic acid	C([C@@H](C)O)(O)=O	Human Class 6	Non-sensitizer
121	124-07-2	Octanoic acid	C(CCCCC)CC(O)=O	Human Class 6	Non-sensitizer
122	8009-03-8	Petrolatum	n/a	Human Class 6	Non-sensitizer
123	108-95-2	Phenol	c1(ccccc1)O	Human Class 6	Non-sensitizer

124	302-79-4	Retinoic acid	C=1(C(CCCC1C)(C)C)\C=C\C(=C\C=C\C(=C\C(O)=O)C)C	Human Class 6	Non-sensitizer
125	69-72-7	Salicylic acid	c1(c(cccc1)O)C(=O)O	Human Class 6	Non-sensitizer
126	151-21-3	Sodium lauryl sulfate	C(CCCCCCCCCCC)COS(=O)(=O)[O-].[Na+]	Human Class 6	Non-sensitizer
127	50-70-4	Sorbitol	O[C@@H]([C@@H]([C@H](CO)O)O)[C@@H](CO)O	Human Class 6	Non-sensitizer
128	59-02-9	Tocopherol	CC(C)CCC[C@@H](C)CCC[C@@H](C)CCC[C@]1(C)CCc2c(C)c(O)c(C)c(C)c2O1	Human Class 6	Non-sensitizer
129	9005-65-6	Tween 80	n/a	Human Class 6	Non-sensitizer
130	1330-20-7	Xylene	n/a	Human Class 6	Non-sensitizer
131	1314-13-2	Zinc oxide	O=[Zn]	Human Class 6	Non-sensitizer

*CASRN 55965-84-9 was used when model input required CASRN. The SMILES string associated with this CASRN was used when model input required a SMILES string.
**CASRN 138-86-3 was used when model input required CASRN. Although there are two CASRN for this chemical, the SMILES string for each CASRN is identical to the other; therefore, this SMILES string was used to represent both CASRN.

Tab. S2: HSDB data set

Number	CAS number	Chemical name	SMILES string	Binary sensitization status
1	9003-01-4	Carbomer	C(C=C)(O)=O	Non-sensitizer
2	111-30-8	Glutaraldehyde	C(CC=O)CC=O	Sensitizer
3	85-44-9	Phthalic anhydride	c12c(C(=O)OC1=O)cccc2	Sensitizer
4	79-07-2	Chloroacetaminde	C(CCl)(N)=O	Sensitizer
5	94-26-8	Butylparaben	c1(ccc(cc1)O)C(=O)OCCCC	Non-sensitizer
6	1163-19-5	Decabromodiphenyl ether	Brc1c(Br)c(Br)c(Oc2c(Br)c(Br)c(Br)c(Br)c2Br)c(Br)c1Br	Non-sensitizer
7	101-20-2	Triclocarban	c1cc(ccc1NC(=O)Nc2ccc(c(c2)Cl)Cl)Cl	Non-sensitizer
8	141-78-6	Ethyl acetate	O(C(=O)C)CC	Non-sensitizer
9	50-00-0	Formaldehyde	C=O	Sensitizer
10	156052-68-5	Zoxamide	CCC(C)(C(=O)CCl)NC(=O)C1=CC(=C(C(=C1)Cl)C)Cl	Sensitizer
11	80-62-6	Methyl methacrylate	C(C(C)=C)(OC)=O	Sensitizer
12	120-47-8	Ethylparaben	c1(C(OCC)=O)ccc(O)cc1	Non-sensitizer
13	141-32-2	Butyl acrylate	C(=O)(OCCCC)C=C	Sensitizer
14	96-33-3	Methyl acrylate	C(=O)(C=C)OC	Sensitizer
15	1861-40-1	Benfluralin	c1(c(cc(C(F)(F)F)cc1[N+](=O)[O-])[N+](=O)[O-])N(CCCC)CC	Sensitizer
16	5124-30-1	Methylene bis(4-cyclohexylisocyanate)	C1CC(CCC1CC2CCC(CC2)N=C=O)N=C=O	Sensitizer
17	2425-06-1	Captafol	ClC(Cl)C(Cl)(Cl)SN1C(=O)[C@H]2CC=CC[C@H]2C1=O	Sensitizer
18	2634-33-5	1,2-Benzisothiazolin-3-one	c1ccc2c(c1)c(=O)[nH]s2	Sensitizer
19	123-54-6	Acetyl acetone	C(CC(C)=O)(C)=O	Sensitizer
20	529-20-4	2-Methylbenzaldehyde	Cc1ccccc1C=O	Non-sensitizer
21	133-06-2	Captan	ClC(Cl)(Cl)SN1C(=O)[C@H]2CC=CC[C@H]2C1=O	Sensitizer
22	87-66-1	Pyrogallic acid	c1(c(cccc1O)O)O	Sensitizer
23	122-60-1	Phenyl glycidyl ether	c1(OC[C@@H]2CO2)ccccc1	Sensitizer
24	120-61-6	Dimethyl terephthalate	c1(C(OC)=O)ccc(C(OC)=O)cc1	Non-sensitizer
25	111-02-4	Squalene	C(=C\CC\C(=C\CC\C=C(\CC\C=C(\CC\C=C(\C)C)C)C)C)(\CC\C=C(\C)C)C	Non-sensitizer
26	164650-44-6	Efinaconazole	C[C@H]([C@](Cn1cncn1)(c2ccc(cc2F)F)O)N3CCC(=C)CC3	Non-sensitizer
27	94-96-2	2-Ethyl-1,3-hexanediol	CCCC(C(CC)CO)O	Non-sensitizer
28	106-89-8	Epichlorohydrin	C1[C@@H](O1)CCl	Sensitizer
29	88-89-1	Picric acid	c1(c(c(cc(c1)[N+](=O)[O-])[N+](=O)[O-])O)[N+](=O)[O-]	Sensitizer
30	4697-36-3	Carbenicillin	CC1([C@@H](N2[C@H](S1)[C@@H](C2=O)NC(=O)C(c3ccccc3)C(=O)O)C(=O)O)C	Sensitizer
31	51630-58-1	Fenvalerate	c1(C(C(=O)OC(c2cc(Oc3ccccc3)ccc2)C#N)C(C)C)ccc(cc1)Cl	Sensitizer
32	2426-08-6	n-Butyl glycidyl ether	C1[C@@H](O1)COCCCC	Sensitizer
33	57-55-6	Propylene glycol	C([C@@H](C)O)O	Non-sensitizer
34	100-43-6	4-Vinylpyridine	c1(ccncc1)C=C	Sensitizer
35	101-68-8	4,4'-Methylenediphenyl diisocyanate	c1cc(ccc1Cc2ccc(cc2)N=C=O)N=C=O	Sensitizer

Number	CAS number	Chemical name	SMILES string	Binary sensitization status
36	479-45-8	Tetryl	c1(c(cc([N+](=O)[O-])cc1[N+](=O)[O-])[N+](=O)[O-])N([N+](=O)[O-])C	Sensitizer
37	68694-11-1	Triflumizole	C(OCCC)/C(=N\c1c(C(F)(F)F)cc(Cl)cc1)n1cncc1	Sensitizer
38	61-72-3	Cloxacillin	Cc1c(c(no1)c2ccccc2Cl)C(=O)N[C@H]3[C@@H]4N(C3=O)[C@H](C(S4)(C)C)C(=O)O	Sensitizer
39	7696-12-0	Tetramethrin	CC(=CC1C(C(=O)OCN2C(=O)C3=C(CCCC3)C2=O)C1(C)C)C	Non-sensitizer
40	35691-65-7	1,2-Dibromo-2,4-dicyanobutane	C(CCC#N)(C#N)(CBr)Br	Sensitizer
41	1675-54-3	Bisphenol A Diglycidyl Ether	C(c1ccc(cc1)OCC1CO1)(c1ccc(cc1)OCC1CO1)(C)C	Sensitizer
42	10124-43-3	Cobaltous sulfate	S(=O)(=O)([O-])[O-].[Co+2]	Sensitizer
43	85-43-8	Tetrahydrophthalic anhydride	C1([C@@H]2[C@@H](C(=O)O1)CC=CC2)=O	Sensitizer
44	7778-50-9	Potassium dichromate	[Cr](O[Cr](=O)(=O)[O-])(=O)(=O)[O-].[K+].[K+]	Sensitizer
45	82-05-3	Benzanthrone	c12c3c4c(cccc4)C(c1cccc2ccc3)=O	Sensitizer
46	99-76-3	Methylparaben	COC(=O)c1ccc(cc1)O	Non-sensitizer
47	101-84-8	Diphenyl ether	c1(Oc2ccccc2)ccccc1	Non-sensitizer
48	142-59-6	Nabam	C(CNC(=S)[S-])NC(=S)[S-].[Na+].[Na+]	Sensitizer
49	100-69-6	2-Vinylpyridine	c1(ccccn1)C=C	Sensitizer
50	140-88-5	Ethyl acrylate	C(=O)(OCC)C=C	Sensitizer
51	79622-59-6	Fluazinam	n1cc(C(F)(F)F)cc(Cl)c1Nc1c([N+]([O-])=O)c(Cl)c(C(F)(F)F)cc1[N+]([O-])=O	Sensitizer
52	131-11-3	Dimethyl phthalate	COC(=O)c1ccccc1C(=O)OC	Sensitizer
53	11051-71-1	Avilamycin	O1[C@@H]([C@]([C@H]2[C@H]([C@@]31O[C@H]1[C@@H](OC(C(C)C)=O)[C@@H](O[C@H]4[C@H]([C@H]([C@H](O[C@H]5[C@H](O)[C@@H](O[C@H]6C[C@@]7([C@@H]([C@H](O6)C)O[C@@]6(O[C@@H]([C@H]([C@@H](C6)O)O[C@@H]6O[C@@H]([C@H]([C@@H](C6)O)OC(=O)c6c(c(c(c(Cl)c6C)O)Cl)OC)C)C)O7)C)[C@@H](OC)[C@H](C)O5)[C@H](O4)COC)O)OC)OC[C@H]1O3)OCO2)(C(=O)C)O)C	Sensitizer
54	94-13-3	Propylparaben	c1c(O)ccc(C(OCCC)=O)c1	Non-sensitizer
55	10141-05-6	Cobaltous nitrate	[N+]([O-])([O-])=O.[N+](=O)([O-])[O-].[Co+2]	Sensitizer
56	60-09-3	Aniline Yellow	c1(\N=N\c2ccccc2)ccc(N)cc1	Sensitizer
57	34590-94-8	Dipropylene glycol monomethyl ether	COCC(C)OCC(C)O	Non-sensitizer
58	8003-22-3	D&C Yellow No. 11	O=C1C(C(=O)c2ccccc12)c3ccc4ccccc4n3	Sensitizer
59	25013-16-5	Butylated hydroxyanisole	[*]C(C)(C)C.COc1ccc(O)cc1	Sensitizer
60	111-40-0	Diethylenetriamine	N(CCN)CCN	Sensitizer
61	108-23-6	Isopropyl chloroformate	C(OC(=O)Cl)(C)C	Sensitizer
62	71-48-7	Cobaltous acetate	[Co+2].CC(=O)[O-].CC(=O)[O-]	Sensitizer
63	106-92-3	Allyl glycidyl ether	C1[C@@H](O1)COCC=C	Sensitizer
64	107-22-2	Glyoxal	C(=O)C=O	Sensitizer
65	112-53-8	1-Dodecanol	C(CCCCCC)CCCCCO	Non-sensitizer
66	1404-04-02	Neomycin	O1[C@H](O[C@H]2[C@H](O[C@H]3[C@@H]([C@@H]([C@H](O3)CO)O[C@@H]3[C@@H]([C@H]([C@@H]([C@@H](O3)CN)O)O)N)O)[C@H]([C@@H](C[C@@H]2N)N)O)[C@@H]([C@H]([C@@H](O)[C@H]1CN)O)N	Sensitizer
67	4170-30-3	Crotonaldehyde	C(=C\C)/C=O	Sensitizer
68	141-43-5	2-Aminoethanol	C(CO)NC	Sensitizer
69	78-18-2	Cyclohexanone peroxide	OOC1(CCCCC1)OOC2(O)CCCCC2	Sensitizer
70	2698-41-1	2-Chlorobenzalmalononitrile	c1(\C=C(\C#N)C#N)c(cccc1)Cl	Sensitizer
71	624-83-9	Methyl isocyanate	CN=C=O	Sensitizer
72	12427-38-2	Maneb	[S-]C(NCCNC([S-])=S)=S.[Mn+2]	Sensitizer
73	107-02-8	Acrolein	C=CC=O	Sensitizer
74	88-73-3	1-Chloro-2-nitrobenzene	c1(c(cccc1)Cl)[N+](=O)[O-]	Sensitizer
75	1334-78-7	Methylbenzaldehydes	c1(ccccc1)C=O.C[*]	Non-sensitizer
76	77-90-7	Acetyl tributyl citrate	CCCCOC(=O)CC(CC(=O)OCCCC)(C(=O)OCCCC)OC(=O)C	Non-sensitizer
77	18454-12-1	Lead chromate oxide	[Cr](=O)(=O)([O-])[O-].O=[Pb].[Pb+2]	Sensitizer
78	5307-02-8	2-Methoxy-1,4-benzenediamine	c1(c(ccc(c1)N)N)OC	Sensitizer
79	5421-46-5	Ammonium thioglycolate	C(CS)(=O)[O-].[NH4+]	Sensitizer

Number	CAS number	Chemical name	SMILES string	Binary sensitization status
80	7446-08-4	Selenium dioxide	[Se](=O)=O	Sensitizer
81	586-62-9	Terpinolene	C1(=C(\C)C)\CCC(C)=CC1	Non-sensitizer
82	4680-78-8	C.I. Acid Green	[Na+].CCN(Cc1cccc(c1)S(=O)(=O)[O-])c2ccc(cc2)C(=C3C=CC(=[N+](CC)Cc4cccc(c4)S(=O)(=O)[O-])C=C3)c5ccccc5	Sensitizer
83	507-70-0	Borneol	CC1(C)[C@@H]2CC[C@@]1(C)[C@@H](O)C2	Non-sensitizer
84	7488-56-4	Selenium disulfide	[Se](=S)=S	Sensitizer
85	112-55-0	1-Dodecanethiol	C(CCCCCC)CCCCCS	Sensitizer
86	123-30-8	4-Aminophenol	c1(ccc(O)cc1)N	Sensitizer
87	26172-55-4	5-Chloro-2-methyl-4-isothiazolin-3-one	CN1SC(=CC1=O)Cl	Sensitizer
88	66230-04-4	Esfenvalerate	CC(C)[C@H](C(=O)O[C@H](C#N)c1cccc(Oc2ccccc2)c1)c3ccc(Cl)cc3	Sensitizer
89	7646-79-9	Cobaltous chloride	[Cl-].[Cl-].[Co+2]	Sensitizer
90	108-32-7	Propylene carbonate	O1[C@@H](COC1=O)C	Non-sensitizer
91	101200-48-0	Tribenuron-methyl	Cc1nc(nc(n1)OC)N(C)C(=O)NS(=O)(=O)c2ccccc2C(=O)OC	Sensitizer
92	7664-93-9	Sulfuric acid	S(=O)(=O)(O)O	Non-sensitizer
93	57-11-4	Stearic acid	C(CCCCCCCCCCC)CCCCCC(=O)O	Non-sensitizer
94	137-26-8	Thiram	CN(C)C(=S)SSC(=S)N(C)C	Sensitizer
95	55297-95-5	Tiamulin	CCN(CC)CCSCC(=O)O[C@@H]1C[C@@](C)(C=C)[C@@H](O)[C@H](C)[C@@]23CC[C@@H](C)[C@@]1(C)[C@@H]2C(=O)CC3	Non-sensitizer
96	63449-39-8	Chlorinated paraffins	CCCC(Cl)CCCC(Cl)CCC(Cl)CCC(Cl)CCC(Cl)CCCC(Cl)CCC	Non-sensitizer
97	4016-14-2	Isopropyl glycidyl ether	C1[C@@H](O1)COC(C)C	Sensitizer
98	97-88-1	n-Butyl methacrylate	C(=O)(OCCCC)C(=C)C	Sensitizer
99	29973-13-5	Ethiofencarb	c1(c(cccc1)OC(=O)NC)CSCC	Sensitizer
100	52-51-7	Bronopol	C(C(CO)([N+](=O)[O-])Br)O	Sensitizer
101	620-23-5	3-Methylbenzaldehyde	c1(cc(ccc1)C)C=O	Non-sensitizer
102	141517-21-7	Trifloxystrobin	C/C(=N\OCc1ccccc1/C(=N/OC)/C(=O)OC)/c2cccc(c2)C(F)(F)F	Sensitizer
103	108-91-8	Cyclohexylamine	C1(CCCCC1)N	Sensitizer
104	104-87-0	4-Methylbenzaldehyde	c1(ccc(cc1)C)C=O	Non-sensitizer
105	75-15-0	Carbon disulfide	C(=S)=S	Sensitizer
106	111-42-2	Diethanolamine	N(CCO)CCO	Sensitizer
107	85-42-7	Hexhydrophthalic anhydride	O1C([C@@H]2CCCC[C@@H]2C1=O)=O	Sensitizer
108	7727-54-0	Ammonium peroxydisulfate	S(OOS(=O)(=O)[O-])(=O)(=O)[O-].[NH4+].[NH4+]	Sensitizer
109	137-66-6	Ascorbyl palmitate	[C@@H]1(OC(=O)C(=C1O)O)[C@H](COC(=O)CCCCCCCCCCCCCCC)O	Non-sensitizer
110	104-55-2	Cinnamaldehyde	c1(ccccc1)/C=C/C=O	Sensitizer
111	96-09-3	Styrene-7,8-oxide	c1([C@@H]2CO2)ccccc1	Sensitizer
112	420-04-2	Cyanamide	C(N)#N	Sensitizer
113	14901-07-6	beta-Ionone	C=1(C(CCCC1C)(C)C)\C=C\C(C)=O	Non-sensitizer
114	5392-40-5	Citral	C(=C\C=O)(/CC/C=C(/C)C)C	Sensitizer
115	126-72-7	Tris(2,3-dibromo-1-propyl)phosphate	P(=O)(OCC(CBr)Br)(OCC(CBr)Br)OCC(CBr)Br	Sensitizer
116	79-10-7	Acrylic acid	C(C=C)(O)=O	Sensitizer
117	129-40-8	1-Chloro-5-nitroanthraquinone	c12c(C(c3c(cccc3C1=O)Cl)=O)cccc2[N+](=O)[O-]	Sensitizer
118	98-86-2	Acetophenone	c1(ccccc1)C(=O)C	Non-sensitizer
119	101-21-3	Chlorpropham	c1(NC(OC(C)C)=O)cc(ccc1)Cl	Sensitizer
120	2655-19-8	Butacarb	CNC(=O)Oc1cc(cc(c1)C(C)(C)C)C(C)(C)C	Sensitizer
121	106-24-1	Geraniol	C(\CC\C=C(\C)C)(=C\CO)C	Non-sensitizer
122	75-07-0	Acetaldehyde	CC=O	Sensitizer
123	99-56-9	4-Nitro-1,2-diaminobenzene	c1(cc(c(N)cc1)N)[N+](=O)[O-]	Sensitizer
124	115-27-5	Chlorendic anhydride	C1([C@@]2([C@@H]3[C@@H](C(=O)OC3=O)[C@@]1(Cl)C(=C2Cl)Cl)Cl)(Cl)Cl	Sensitizer
125	108-24-7	Acetic anhydride	C(OC(=O)C)(C)=O	Sensitizer
126	15972-60-8	Alachlor	c1(N(C(CCl)=O)COC)c(CC)cccc1CC	Sensitizer
127	123-86-4	n-Butyl acetate	O(CCCC)C(C)=O	Non-sensitizer
128	111-70-6	1-Heptanol	C(CCCO)CCC	Non-sensitizer
129	112-92-5	1-Octadecanol	C(CCCCCCCCC)CCCCCCCCO	Sensitizer

Number	CAS number	Chemical name	SMILES string	Binary sensitization status
130	110-85-0	Piperazine	C1CNCCN1	Sensitizer
131	123-77-3	1,1'-Azobis(formamide)	C(=O)(/N=N/C(=O)N)N	Sensitizer
132	541-73-1	1,3-Dichlorobenzene	c1c(cccc1Cl)Cl	Non-sensitizer
133	22967-92-6	Methylmercury cation	[CH3-][Hg+2]	Sensitizer
134	79-22-1	Methyl chloroformate	C(OC)(=O)Cl	Sensitizer
135	92-52-4	Biphenyl	c1(c2ccccc2)ccccc1	Sensitizer
136	26447-14-3	Cresyl glycidyl ether	c1(OC[C@@H]2CO2)ccccc1.C*	Sensitizer
137	129-35-1	1-Chloro-2-methylanthraquinone	c12c(C(c3ccccc3C1=O)=O)ccc(c2Cl)C	Sensitizer
138	103-11-7	2-Ethylhexyl acrylate	C([C@@H](CCCC)CC)OC(C=C)=O	Non-sensitizer
139	142-91-6	Isopropyl palmitate	C(=O)(OC(C)C)CCCCCCCCCCCCCCC	Non-sensitizer
140	133-07-3	Flopet	c1ccc2c(c1)C(=O)N(C2=O)SC(Cl)(Cl)Cl	Sensitizer
141	26523-78-4	Tris(nonylphenyl)phosphite	CCCCCCCCCc1ccccc1OP(Oc2ccccc2CCCCCCCCC)Oc3ccccc3CCCCCCCCC	Sensitizer
142	334-48-5	Decanoic acid	C(CCCCCC)CCC(=O)O	Non-sensitizer
143	174671-46-6	Tavaborole	c1c(ccc2B(OCc12)O)F	Non-sensitizer
144	110-43-0	2-Heptanone	C(CCCC)C(C)=O	Non-sensitizer
145	818-61-1	2-Hydroxyethyl acrylate	C(OCCO)(C=C)=O	Sensitizer
146	5989-27-5	D-Limonene	C([C@@H]1CCC(C)=CC1)(C)=C	Sensitizer
147	107-41-5	2-Methyl-2,4-pentanediol	C(C[C@@H](C)O)(C)(C)O	Non-sensitizer
148	75-21-8	Ethylene oxide	C1CO1	Sensitizer
149	3761-53-3	Ponceau MX	Cc1ccc(c(c1)C)/N=N/c2c3ccc(cc3cc(c2O)S(=O)(=O)[O-])S(=O)(=O)[O-].[Na+].[Na+]	Sensitizer
150	100-47-0	Benzonitrile	c1(ccccc1)C#N	Non-sensitizer
151	123-15-9	2-Methylpentaldehyde	C(CCC)(C=O)C	Sensitizer
152	5323-95-5	Sodium ricinoleate	[Na+].CCCCCC[C@@H](O)C\C=C/CCCCCCCC(=O)[O-]	Sensitizer
153	1918-02-1	Picloram	c1(c(c(c(Cl)c(n1)Cl)N)Cl)C(O)=O	Non-sensitizer
154	1403-66-3	Gentamicin	O([C@@H]1[C@@H]([C@@H](O[C@@H]2O[C@@H](CC[C@@H]2N)[C@@H](NC)C)[C@@H](N)C[C@@H]1N)O)[C@@H]1[C@@H]([C@@H]([C@@](C)(O)CO1)NC)O	Non-sensitizer
155	98-01-1	Furfural	c1(ccco1)C=O	Sensitizer
156	150-75-4	N-Methyl-4-aminophenol	c1(ccc(O)cc1)NC	Sensitizer
157	302-01-2	Hydrazine	NN	Sensitizer
158	2425-85-6	C.I. Pigment Red 3	c12c(\N=N\c3c(cc(C)cc3)[N+](=O)[O-])c(ccc1cccc2)O	Sensitizer
159	10124-48-8	Mercuric ammonium chloride	[NH2-].[Cl-].[Hg+2]	Sensitizer
160	142-64-3	Piperazine hydrochloride	C1CNCCN1.Cl.Cl	Sensitizer
161	100-97-0	Methenamine	C1N2CN3CN(CN1C3)C2	Sensitizer
162	30525-89-4	Paraformaldehyde	C=O	Sensitizer
163	128-66-5	C.I. Vat Yellow 4	c12c3c4c5c(cccc5)C(c3ccc1c1c(cccc1)C(c2cc4)=O)=O	Sensitizer
164	2832-40-8	C.I. Disperse Yellow 3	c1(\N=N\c2ccc(NC(C)=O)cc2)c(ccc(c1)C)O	Sensitizer
165	842-07-9	C.I. Solvent Yellow 14	c12c(\N=N\c3ccccc3)c(ccc1cccc2)O	Sensitizer
166	101-90-6	Diglycidyl resorcinol ether	c1cc(cc(c1)OCC2CO2)OCC3CO3	Sensitizer
167	111-27-3	1-Hexanol	CCCCCCO	Non-sensitizer
168	765-34-4	Glycidaldehyde	C1(CO1)C=O	Sensitizer
169	590-00-1	Potassium sorbate	C(=C\C=C\C)\C(=O)[O-].[K+]	Non-sensitizer
170	123-31-9	Hydroquinone	c1(ccc(cc1)O)O	Sensitizer
171	79-94-7	2,2',6,6'-Tetrabromobisphenol A	C(c1cc(c(O)c(c1)Br)Br)(c1cc(c(O)c(c1)Br)Br)(C)C	Non-sensitizer
172	81-88-9	Rhodamine B	c1(c2c(oc3c1ccc(\c3)=[N+](/CC)CC)cc(N(CC)CC)cc2)c1c(cccc1)C(O)=O.[ClH-]	Sensitizer
173	78-93-3	Methyl ethyl ketone	C(CC)(C)=O	Non-sensitizer
174	7789-00-6	Potassium chromate	[Cr](=O)(=O)([O-])[O-].[K+].[K+]	Sensitizer
175	25322-69-4	Polypropylene glycol	O([C@@H](CO)C)C[C@@H](C)O	Non-sensitizer
176	7647-10-1	Palladium(2+) chloride	[Pd](Cl)Cl	Sensitizer
177	72-14-0	Sulfathiazole	Nc1ccc(cc1)S(=O)(=O)Nc2nccs2	Sensitizer
178	60-12-8	2-Phenylethanol	c1(ccccc1)CCO	Non-sensitizer
179	107-21-1	Ethylene glycol	C(CO)O	Sensitizer
180	16071-86-6	C.I. Direct Brown 95	c1(c2ccc(\N=N\c3cc(c(O)cc3)C(=O)[O-])cc2)ccc(\N=N\c2c(c(\N=N\c3cc(ccc3[O-])S(=O)(=O)[O-])ccc2O)[O-])cc1.[Cu+2].[Na+].[Na+]	Sensitizer
181	121-73-3	1-Chloro-3-nitrobenzene	c1(cc(ccc1)Cl)[N+](=O)[O-]	Sensitizer
182	101-99-5	Ethyl-n-phenylcarbamate	CCOC(=O)Nc1ccccc1	Sensitizer

Number	CAS number	Chemical name	SMILES string	Binary sensitization status
183	25584-83-2	Hydroxypropyl acrylate	C(=O)(OCCCO)C=C	Sensitizer
184	104-91-6	4-Nitrosophenol	c1(ccc(O)cc1)N=O	Sensitizer
185	106-68-3	3-Octanone	C(CCCC)C(=O)CC	Non-sensitizer
186	97-63-2	Ethyl methacrylate	C(OCC)(C(C)=C)=O	Sensitizer
187	93-58-3	Methyl benzoate	COC(=O)c1ccccc1	Non-sensitizer
188	127-91-3	beta-Pinene	CC1(C)[C@H]2CCC(=C)[C@@H]1C2	Sensitizer
189	78-70-6	Linalool	C([C@@](C=C)(C)O)C\C=C(\C)C	Non-sensitizer
190	120-80-9	Catechol	c1(c(cccc1)O)O	Sensitizer
191	107-96-0	2-Mercaptopropionic acid	C(C(=O)O)CS	Non-sensitizer
192	112-05-0	Nonanoic acid	C(CCCCC)CCC(=O)O	Non-sensitizer
193	111-54-6	Ethylenebisdithiocarbamic acid	C(CNC(=S)S)NC(=S)S	Sensitizer
194	99-49-0	Carvone	CC(=C)C1CC=C(C)C(=O)C1	Sensitizer
195	8003-34-7	Pyrethrum	n/a	Non-sensitizer
196	121-21-1	Pyrethrin I	CC(=C[C@@H]1[C@@H](C(=O)O[C@H]2CC(=O)C(=C2C)C\C=C/C=C)C1(C)C)C	Non-sensitizer
197	78-11-5	Pentaerythritol tetranitrate	C(CO[N+](=O)[O-])(CO[N+](=O)[O-])(CO[N+](=O)[O-])CO[N+](=O)[O-]	Non-sensitizer
198	78-94-4	Methyl vinyl ketone	C(C=C)(C)=O	Sensitizer
199	3319-31-1	Tri(2-ethylhexyl) trimelliate	CCCCC(CC)COC(=O)c1ccc(c(c1)C(=O)OCC(CC)CCCC)C(=O)OCC(CC)CCCC	Non-sensitizer
200	2238-07-5	Diglycidyl ether	C([C@@H]1CO1)OC[C@@H]1CO1	Sensitizer
201	95-70-5	2-Methyl-1,4-benzenediamine	Cc1cc(ccc1N)N	Non-sensitizer
202	3118-97-6	C.I. Solvent Orange 7	Cc1ccc(N=Nc2c(O)ccc3ccccc23)c(C)c1	Sensitizer
203	121-29-9	Pyrethrin II	COC(=O)\C(=C\[C@@H]1[C@@H](C(=O)O[C@H]2CC(=O)C(=C2C)C\C=C/C=C)C1(C)C)\C	Non-sensitizer
204	334-88-3	Diazomethane	[N+](=C)=[N-]	Sensitizer
205	64-00-6	Phenol,3(1-methylethyl)-, methyl carbamate	c1(cc(ccc1)C(C)C)OC(=O)NC	Sensitizer
206	10588-01-9	Sodium dichromate	[Cr](O[Cr](=O)(=O)[O-])(=O)(=O)[O-].[Na+].[Na+]	Sensitizer
207	533-74-4	Dazomet	CN1CN(C(=S)SC1)C	Sensitizer
208	23135-22-0	Oxamyl	CNC(=O)ON=C(SC)C(=O)N(C)C	Sensitizer
209	14484-64-1	Ferbam	CN(C)C1=S[Fe+3]23([SH-]C(=S2)N(C)C)([SH-]C(=S3)N(C)C)[SH-]1	Sensitizer
210	25155-30-0	Sodium dodecylbenzenesulfonate	c1(c(cccc1)CCCCCCCCCCCC)S(=O)(=O)[O-].[Na+]	Sensitizer
211	64-18-6	Formic acid	C(=O)O	Sensitizer
212	34681-10-2	Butocarboxim	CNC(=O)ON=C(C)C(C)SC	Sensitizer
213	26248-24-8	Sodium tridecylbenzene sulfonate	c1(ccc(S(=O)(=O)[O-])cc1)CCCCCCCCCCCCC.[Na+]	Sensitizer
214	65907-30-4	Furathiocarb	c12c(OC(=O)N(SN(C(=O)OCCCC)C)C)cccc1CC(O2)(C)C	Sensitizer
215	1338-41-6	Sorbitan monostearate	CCCCCCCCCCCCCCCCCC(=O)OC[C@@H](O)[C@H]1OC[C@H](O)[C@H]1O	Sensitizer
216	51218-45-2	Metolachlor	c1(N([C@@H](COC)C)C(CCl)=O)c(cccc1C)CC	Sensitizer
217	1967-16-4	Chlorbufam	c1(NC(O[C@@H](C#C)C)=O)cc(ccc1)Cl	Sensitizer
218	82-68-8	Pentachloronitrobenzene	c1(c(c(c(Cl)c(c1Cl)Cl)Cl)Cl)[N+](=O)[O-]	Sensitizer
219	26644-46-2	Triforine	C(N1CCN(CC1)C(C(Cl)(Cl)Cl)NC=O)(C(Cl)(Cl)Cl)NC=O	Sensitizer
220	90-43-7	o-Phenylphenol	c1(c2c(cccc2)O)ccccc1	Non-sensitizer
221	1918-18-9	Swep	c1(cc(c(Cl)cc1)Cl)NC(OC)=O	Sensitizer
222	12122-67-7	Zineb	C(CNC(=S)[S-])NC1=S[Zn+2][SH-]1	Non-sensitizer
223	75-02-5	Vinyl fluoride	C(=C)F	Non-sensitizer
224	25402-06-6	Cinerin I	C\C=C/CC1=C(C)[C@H](CC1=O)OC(=O)[C@@H]2[C@@H](C=C(C)C)C2(C)C	Non-sensitizer
225	121-20-0	Cinerin II	COC(=O)\C(=C\[C@@H]1[C@@H](C(=O)O[C@H]2CC(=O)C(=C2C)C\C=C/C)C1(C)C)\C	Non-sensitizer
226	90982-32-4	Chlorimuron-ethyl	CCOC(=O)c1ccccc1S(=O)(=O)NC(=O)Nc2nc(cc(n2)Cl)OC	Non-sensitizer
227	138-86-3	Limonene	C([C@@H]1CCC(C)=CC1)(C)=C	Non-sensitizer
228	112-60-7	Tetraethylene glycol	O(CCOCCO)CCOCCO	Non-sensitizer
229	7787-47-5	Beryllium chloride	[Be](Cl)Cl	Sensitizer
230	19044-88-3	Oryzalin	c1(c(cc(S(N)(=O)=O)cc1[N+](=O)[O-])[N+](=O)[O-])N(CCC)CCC	Sensitizer
231	556-52-5	Glycidol	C1(CO1)CO	Sensitizer

Number	CAS number	Chemical name	SMILES string	Binary sensitization status
232	89-32-7	Pyromellitic dianhydride	c12c(c(=O)oc1=O)cc1c(=O)oc(c1c2)=O	Sensitizer
233	503-38-8	Diphosgene	C(OC(=O)Cl)(Cl)(Cl)Cl	Sensitizer
234	110-02-1	Thiophene	c1ccsc1	Sensitizer
235	9003-73-0	Polylimonene	C([C@@H]1CCC(C)=CC1)(C)=C	Sensitizer
236	81-48-1	Alizurol purple	c12c(C(=O)c3c(C2=O)cccc3)c(ccc1Nc1ccc(cc1)C)O	Sensitizer
237	94-36-0	Benzoyl peroxide	c1(C(OOC(c2ccccc2)=O)=O)ccccc1	Sensitizer
238	34681-23-7	Butoxycarboxim	CNC(=O)ON=C(C)C(C)S(=O)(=O)C	Sensitizer
239	12108-13-3	Methylcyclopentadienyl manganese tricarbonyl	[C-]1(C=CC=C1)C.[O+]#[C-].[O+]#[C-].[O+]#[C-].[Mn+]	Non-sensitizer
240	2451-62-9	Triglycidyl isocyanurate	n1(c(n(c(=O)n(c1=O)C[C@@H]1CO1)C[C@@H]1CO 1)=O)C[C@@H]1CO1	Sensitizer
241	80-48-8	Methyl p-methylbenzenesulfonate	S(c1ccc(C)cc1)(OC)(=O)=O	Sensitizer
242	94-09-7	Benzocaine	c1cc(C(OCC)=O)ccc1N	Sensitizer
243	87-56-9	Mucochloric acid	C(=O)/C(=C(\C(=O)O)/Cl)/Cl	Sensitizer
244	84-65-1	Anthraquinone	c12c(C(c3ccccc3C1=O)=O)cccc2	Sensitizer
245	461-58-5	Cyanoguanidine	NC(=N)NC#N	Non-sensitizer
246	5466-77-3	Octinoxate	CCCCC(CC)COC(=O)C=Cc1ccc(OC)cc1	Non-sensitizer
247	51-78-5	4-Aminophenol hydrochloride	c1(ccc(O)cc1)N.Cl	Sensitizer
248	541-41-3	Ethyl chloroformate	O(C(=O)Cl)CC	Sensitizer
249	106-50-3	1,4-Benzenediamine	c1cc(ccc1N)N	Sensitizer
250	624-49-7	Dimethyl fumarate	C(\C=C\C(OC)=O)(OC)=O	Sensitizer
251	89-61-2	1,4-Dichloro-2-nitrobenzene	c1(c(ccc(c1)Cl)Cl)[N+](=O)[O-]	Sensitizer
252	6846-50-0	2,2,4-Trimethyl-1,3-pentanediol diisobutyrate	C([C@@H](OC(C(C)C)=O)C(C)C)(COC(C(C)C)=O)(C)C	Non-sensitizer
253	79-11-8	Chloroacetic acid	C(CCl)(O)=O	Sensitizer
254	1303-28-2	Arsenic pentoxide	[As](=O)(=O)O[As](=O)=O	Sensitizer
255	78-51-3	Tris(2-butoxyethyl) phosphate	P(=O)(OCCOCCCC)(OCCOCCCC)OCCOCCCC	Non-sensitizer
256	82-44-0	1-Chloroanthraquinone	c12c(C(c3ccccc3C1=O)=O)cccc2Cl	Sensitizer
257	117-11-3	1-Amino-5-chloroanthraquinone	c12c(C(c3c(cccc3C1=O)Cl)=O)cccc2N	Sensitizer
258	110-66-7	Amyl mercaptan	C(CCS)CC	Sensitizer
259	75567-37-2	Ingenol mebutate	C1=C([C@@H]([C@@]2([C@@]31[C@@H](C[C@@ H]1[C@H]([C@H](C=C([C@H]2O)CO)C3=O)[C@]1(C)C)C)O)OC(=O)/C(=C\C)C)C	Non-sensitizer
260	64-17-5	Ethanol	CCO	Sensitizer
261	62-53-3	Aniline	c1ccc(cc1)N	Sensitizer
262	106-25-2	Nerol	C(=C\CO)(\CC/C=C(/C)C)C	Non-sensitizer
263	2682-20-4	Methylisothiazolinone	Cn1c(=O)ccs1	Sensitizer
264	106-23-0	Citronellal	C(CC/C=C(/C)C)(CC=O)C	Non-sensitizer
265	110-27-0	Isopropyl myristate	C(CCCCCCCCCCCC)C(=O)OC(C)C	Non-sensitizer
266	488-10-8	Jasmone	CC\C=C/CC1=C(C)CCC1=O	Non-sensitizer
267	1490-04-6	Menthol	C1[C@@H]([C@@H](C[C@@H](C)C1)O)C(C)C	Non-sensitizer
268	55-56-1	Chlorhexidine	c1(NC(NC(NCCCCCCNC(NC(Nc2ccc(Cl)cc2)=N)=N)= N)=N)ccc(Cl)cc1	Sensitizer
269	4080-31-3	N-(3-Chloroallyl)hexaminium chloride	[N+]12(CN3CN(C1)CN(C2)C3)C\C=C\Cl.[ClH-]	Non-sensitizer
270	68603-42-9	Coconut diethanolamide	n/a	Non-sensitizer
271	65277-42-1	Ketoconazole	CC(=O)N1CCN(CC1)c2ccc(cc2)OC[C@@H]3CO[C@ @](O3)(Cn4ccnc4)c5ccc(cc5Cl)Cl	Sensitizer
272	59-87-0	Nitrofurazone	c1(oc(cc1)/C=N/NC(=O)N)[N+](=O)[O-]	Sensitizer
273	121-33-5	Vanillin	c1(cc(ccc1O)C=O)OC	Non-sensitizer
274	109-16-0	Triethylene glycol dimethacrylate	O(C(C(C)=C)=O)CCOCCOCCOC(C(C)=C)=O	Sensitizer
275	26761-40-0	Diisodecyl phthalate	CC(C)CCCCCCCOC(=O)c1ccccc1C(=O)OCCCCCCC C(C)C	Non-sensitizer
276	26027-38-3	Nonoxynols	c1(ccc(OCCO)cc1)CCCCCCCCC	Non-sensitizer
277	110-97-4	Diisopropanolamine	C([C@@H](C)O)NC[C@@H](C)O	Non-sensitizer
278	811-97-2	1,1,1,2-Tetrafluoroethane	C(CF)(F)(F)F	Non-sensitizer
279	2835-95-2	5-Amino-o-cresol	c1(c(ccc(c1)N)C)O	Sensitizer
280	21087-64-9	Metribuzin	CC(C)(C)c1c(=O)n(c(nn1)SC)N	Non-sensitizer

Number	CAS number	Chemical name	SMILES string	Binary sensitization status
281	564-25-0	Doxycycline	C[C@H]1c2cccc(c2C(=O)C3=C([C@]4([C@@H]([C@H]([C@H]13)O)[C@@H](C(=C(C4=O)C(=O)N)O)N(C)C)O)O)O	Sensitizer
282	105-55-5	N,N'-Diethylthiourea	C(NCC)(NCC)=S	Sensitizer
283	150-13-0	4-Aminobenzoic acid	c1cc(ccc1C(=O)O)N	Sensitizer
284	8001-54-5	Benzalkonium chloride compounds	c1(C[N+](C)(C)*)ccccc1.[ClH-]	Sensitizer
285	100-51-6	Benzyl alcohol	OCc1ccccc1	Non-sensitizer
286	97-64-3	Ethyl lactate	CCOC(=O)C(C)O	Non-sensitizer
287	111-77-3	Diethylene glycol monomethyl ether	C(OCCO)COC	Non-sensitizer
288	28553-12-0	Diisononyl phthalate	CCCCCCC(C)COC(=O)c1ccccc1C(=O)OCC(C)CCCCCC	Non-sensitizer
289	92-87-5	Benzidine	c1(c2ccc(N)cc2)ccc(N)cc1	Sensitizer
290	7446-34-6	Selenium sulfide	S=[Se]	Sensitizer
291	102-76-1	Triacetin	C(COC(C)=O)(COC(C)=O)OC(C)=O	Non-sensitizer
292	55283-68-6	Ethalfluralin	c1(c(cc(C(F)(F)F)cc1[N+](=O)[O-])[N+](=O)[O-])N(CC(C)=C)CC	Sensitizer
293	110-19-0	Isobutyl acetate	C(COC(=O)C)(C)C	Non-sensitizer
294	6440-58-0	1,3-Dimethylol-5,5-dimethylhydantoin	CC1(C(=O)N(C(=O)N1CO)CO)C	Sensitizer
295	101-54-2	p-Aminodiphenylamine	c1(Nc2ccccc2)ccc(N)cc1	Sensitizer
296	124-07-2	Octanoic acid	C(CCCCC)CC(O)=O	Non-sensitizer
297	137-30-4	Ziram	C(=S)(N(C)C)[S-].[S-]C(N(C)C)=S.[Zn+2]	Sensitizer
298	54-64-8	Thimerosal	c1(c(cccc1)C(=O)[O-])S[Hg]CC.[Na+]	Sensitizer
299	104-61-0	Dihydro-5-pentyl-2(3H)-furanone	CCCCCC1CCC(=O)O1	Non-sensitizer
300	79-57-2	Oxytetracycline	C[C@]1(c2cccc(c2C(=O)C3=C([C@]4([C@@H]([C@H]([C@@H]31)O)[C@@H](C(=C(C4=O)C(=O)N)O)N(C)C)O)O)O)O)O	Sensitizer
301	868-77-9	2-Hydroxyethyl methacrylate	C(=O)(C(=C)C)OCCO	Sensitizer
302	92-48-8	Methyl coumarin	c12c(oc(=O)cc2)ccc(c1)C	Non-sensitizer
303	55-65-2	Guanethidine	N1(CCNC(=N)N)CCCCCCC1	Sensitizer
304	505-60-2	Bis(2-chloroethyl)sulfide	S(CCCl)CCCl	Sensitizer
305	95-48-7	o-Cresol	c1(c(cccc1)O)C	Sensitizer
306	63-25-2	Carbaryl	c12c(OC(NC)=O)cccc1cccc2	Sensitizer
307	81-14-1	Musk ketone	c1(c(c(c(C(C)=O)c(c1[N+](=O)[O-])C)C)[N+](=O)[O-])C(C)(C)C	Non-sensitizer
308	21245-02-3	Padimate O	c1(C(=O)OCC(CCCC)CC)ccc(cc1)N(C)C	Sensitizer
309	95-95-4	2,4,5-Trichlorophenol	c1c(c(cc(c1Cl)Cl)Cl)O	Non-sensitizer
310	69430-24-6	Cyclomethicone	[Si](O*)(*)(C)C	Non-sensitizer
311	126-73-8	Tributyl phopshate	CCCCOP(=O)(OCCCC)OCCCC	Non-sensitizer
312	80-08-0	Dapsone	S(c1ccc(N)cc1)(c1ccc(N)cc1)(=O)=O	Sensitizer
313	105-54-4	Ethyl n-butyrate	C(=O)(CCC)OCC	Non-sensitizer
314	122-97-4	3-Phenylpropanol	c1(ccccc1)CCCO	Non-sensitizer
315	106-22-9	Citronellol	C(CC/C=C(/C)C)(CCO)C	Non-sensitizer
316	131-74-8	Ammonium picrate	c1(c(c(cc(c1)[N+](=O)[O-])[N+](=O)[O-])[O-])[N+](=O)[O-].[NH4+]	Sensitizer
317	75-91-2	tert-Butyl hydroperoxide	C(OO)(C)(C)C	Sensitizer
318	99-87-6	p-Cymene	c1(C(C)C)ccc(C)cc1	Non-sensitizer
319	111-90-0	Diethylene glycol monomethyl ether	C(COCC)OCCO	Non-sensitizer
320	684-93-5	N-nitroso-n-methylurea	CN(C(=O)N)N=O	Sensitizer
321	12002-03-8	Paris Green	CC(=O)[O-].CC(=O)[O-].[O-][As]1O[As](O[As](O1)[O-])[O-].[O-][As]1O[As](O[As](O1)[O-])[O-].[Cu+2].[Cu+2].[Cu+2].[Cu+2]	Sensitizer
322	542-92-7	1,3-Cyclopentadiene	C1C=CC=C1	Sensitizer
323	123-62-6	Propionic anhydride	O(C(CC)=O)C(CC)=O	Sensitizer
324	85-68-7	Butyl benzyl phthalate	CCCCOC(=O)c1ccccc1C(=O)OCc2ccccc2	Non-sensitizer
325	1222-05-5	Galaxolide	O1C[C@@H](c2c(cc3c(C([C@@H](C)C3(C)C)(C)C)c2)C1)C	Non-sensitizer
326	5471-51-2	Raspberry ketone	CC(=O)CCc1ccc(cc1)O	Non-sensitizer
327	105-60-2	Caprolactam	C1(CCCCCN1)=O	Sensitizer
328	108-78-1	Melamine	c1(nc(nc(n1)N)N)N	Non-sensitizer
329	35367-38-5	Diflubenzuron	c1(C(=O)NC(=O)Nc2ccc(cc2)Cl)c(cccc1F)F	Non-sensitizer

Number	CAS number	Chemical name	SMILES string	Binary sensitization status
330	79538-32-2	Tefluthrin	Cc1c(c(c(c(c1F)F)COC(=O)[C@H]2[C@H](C2(C)C)/C=C(/C(F)(F)F)\Cl)F)F	Non-sensitizer
331	25956-17-6	Allura red ac dye	c12c(cc(cc2)S(=O)(=O)[O-])ccc(c1/N=N/c1c(cc(c(c1)C)S(=O)(=O)[O-])OC)O.[Na+].[Na+]	Non-sensitizer
332	563-80-4	Methyl isopropyl ketone	C(C(C)=O)(C)C	Non-sensitizer
333	106-31-0	Butyric anhydride	C(=O)(OC(=O)CCC)CCC	Sensitizer
334	121-32-4	Ethyl vanillin	CCOc1cc(ccc1O)C=O	Non-sensitizer
335	143-08-8	1-Nonanol	CCCCCCCCCO	Non-sensitizer
336	13454-96-1	Platinum tetrachloride	[Pt](Cl)(Cl)(Cl)Cl	Sensitizer
337	107-46-0	Hexamethyldisiloxane	[Si](O[Si](C)(C)C)(C)(C)C	Non-sensitizer
338	111-13-7	2-Octanone	C(CC(=O)C)CCCC	Non-sensitizer
339	94-59-7	Safrole	c12c(ccc(c1)CC=C)OCO2	Non-sensitizer
340	2216-51-5	(L)-Menthol	C1[C@H]([C@@H](C[C@@H](C)C1)O)C(C)C	Non-sensitizer
341	10025-65-7	Platinous chloride	[Pt](Cl)Cl	Sensitizer
342	39300-45-3	Dinocap	c1(c(cccc1)[N+](=O)[O-])OC(\C=C\C)=O.C(CCCC)C[C@@H](C)*.[N+](=O)(*)[O-]	Sensitizer
343	6317-18-6	Methylene thiocyanate	C(SC#N)SC#N	Non-sensitizer
344	138-89-6	N,N-Dimethyl-p-nitrosoaniline	c1(ccc(N=O)cc1)N(C)C	Sensitizer
345	148-79-8	Thiabendazole	c1ccc2c(c1)[nH]c(n2)c3cscn3	Non-sensitizer
346	36653-82-4	1-Hexadecanol	CCCCCCCCCCCCCCCCO	Non-sensitizer
347	26571-11-9	Nonoxynol-9	c1(ccc(cc1)CCCCCCCCC)OCCOCCOCCOCCOCCOCCOCCOCCOCCO	Sensitizer
348	77-09-8	Phenolphthalein	C1(c2c(cccc2)C(O1)=O)(c1ccc(O)cc1)c1ccc(O)cc1	Sensitizer
349	101-81-5	1,1'-Methylenebibenzene	c1(Cc2ccccc2)ccccc1	Non-sensitizer
350	60-00-4	Ethylenediamine tetraacetic acid	N(CCN(CC(O)=O)CC(O)=O)(CC(O)=O)CC(O)=O	Sensitizer
351	21593-23-7	Cepharin	N12[C@@H]([C@@H](NC(CSc3ccncc3)=O)C2=O)SCC(=C1C(O)=O)COC(C)=O	Sensitizer
352	81-21-0	Dicyclopentadiene dioxide	C1[C@@H]2[C@@H]3[C@@H]([C@@H]1[C@@H]1[C@@H]2O1)C[C@@H]1[C@@H]3O1	Non-sensitizer
353	98-59-9	p-Toluenesulfonyl chloride	S(=O)(=O)(c1ccc(cc1)C)Cl	Sensitizer
354	96-08-2	1,2:8,9-Diepoxy-p-menthane	C12C(O1)(CCC(C2)C1C(O1)C)C	Non-sensitizer
355	104-93-8	1-Methoxy-4-methylbenzene	COc1ccc(C)cc1	Non-sensitizer
356	121-39-1	Ethyl-2,3-epoxy-3-phenylpropionate	C1(C(O1)C(=O)OCC)c1ccccc1	Non-sensitizer
357	140-67-0	1-Methoxy-4-(2-propenyl)benzene)	c1(ccc(OC)cc1)CC=C	Non-sensitizer
358	409-02-9	Methylheptenone	C(=C\CCC(=O)C)(/C)C	Non-sensitizer
359	103-64-0	beta-Bromostyrene	c1(ccccc1)/C=C/Br	Non-sensitizer
360	54-42-2	Idoxuridine	OC[C@H]1O[C@H](C[C@@H]1O)N2C=C(I)C(=O)NC2=O	Sensitizer
361	589-18-4	4-Methylbenzyl alcohol	Cc1ccc(CO)cc1	Non-sensitizer
362	8002-75-3	Palm Oil (From Fruit)	n/a	Non-sensitizer
363	12656-85-8	C.I. Pigment Red 104	n/a	Sensitizer
364	7440-06-4	Platinum	[Pt]	Sensitizer
365	9006-65-9	Dimethicone	n/a	Non-sensitizer
366	8006-64-2	Turpentine	n/a	Sensitizer
367	68647-73-4	Tea tree oil	n/a	Sensitizer
368	25265-71-8	Dipropylene glycol	n/a	Non-sensitizer
369	8000-27-9	Cedarwood oil	n/a	Non-sensitizer
370	8008-57-9	Oil of Orange	n/a	Non-sensitizer
371	8000-42-8	Oil of Caraway	n/a	Non-sensitizer
372	7440-02-0	Nickel, elemental	[Ni]	Sensitizer
373	8015-64-3	Angelica oils	n/a	Non-sensitizer
374	8016-23-7	Guaiac wood oil	n/a	Non-sensitizer
375	8006-84-6	Fennel oil	n/a	Sensitizer

Tab. S3: Raw numbers for global performance metrics

Model	Number of chemicals entered into model (n)	Number of predictions returned – base settings (n)					Number of predictions returned – optimized settings (n)				
		TP	TN	FP	FN	Total	TP	TN	FP	FN	Total
**Basketter et al. (2014)**											
PredSkin	123	91	10	10	12	123	54	1	7	1	63
Toxtree*	123	69	16	4	34	123	n/a	n/a	n/a	n/a	n/a
QSAR Toolbox**	123	53	5	6	5	69	53	5	6	5	69
REACHAcross™	131	75	13	7	28	123	34	8	1	10	53
Danish QSAR Database	131	41	13	3	28	85	33	9	3	24	69
CAESAR	123	67	8	11	20	106	47	5	1	10	63
TIMES-SS	123	38	17	1	57	113	61	11	0	20	92
Derek	123	85	17	3	17	122	85	17	3	13	118
**HSDB**											
PredSkin	361	206	37	94	24	361	113	1	26	0	140
Toxtree*	361	127	85	46	103	361	n/a	n/a	n/a	n/a	n/a
QSAR Toolbox**	361	103	34	39	17	193	103	34	39	17	193
REACHAcross™	375	169	78	52	57	356	75	37	18	10	140
Danish QSAR Database	375	98	81	20	43	242	75	72	14	28	189
CAESAR	361	138	34	89	51	312	44	14	42	5	105
TIMES-SS	361	98	107	19	104	328	59	41	19	18	137
Derek	361	154	85	44	72	355	154	78	44	50	326

TP: true positive; TN: true negative; FP: false positive; FN: false negative
* Toxtree is a structural alert model and, therefore, does not have any optimization features.
**For QSAR Toolbox, both the base and optimized settings have the same numbers because the automated batch mode applies the same settings universally.

Tab. S4: Model sensitivity and specificity for high confidence results

Model	Sensitivity (%)	Specificity (%)
**Basketter et al. (2014)**		
PredSkin	98	13
Toxtree	67	80
QSAR Toolbox	91	45
REACHAcross™	77	89
Danish QSAR Database	58	75
CAESAR	82	83
TIMES-SS	75	100
Derek	87	85
**HSDB**		
PredSkin	100	4
Toxtree	55	65
QSAR Toolbox	86	47
REACHAcross™	88	67
Danish QSAR Database	73	84
CAESAR	90	25
TIMES-SS	77	68
Derek	75	64

Tab. S5: Model false predictions for high confidence results

Model	False positive (%)	False negative (%)	False predictions (%)
**Basketter et al. (2014)**			
PredSkin	88	2	13
Toxtree	20	33	31
QSAR Toolbox	55	9	16
REACHAcross™	11	23	21
Danish QSAR Database	25	42	39
CAESAR	17	18	17
TIMES-SS	0	25	22
Derek	15	13	14
**HSDB**			
PredSkin	96	0	19
Toxtree	35	45	41
QSAR Toolbox	53	14	29
REACHAcross™	33	12	20
Danish QSAR Database	16	27	22
CAESAR	75	10	45
TIMES-SS	32	23	27
Derek	36	25	29

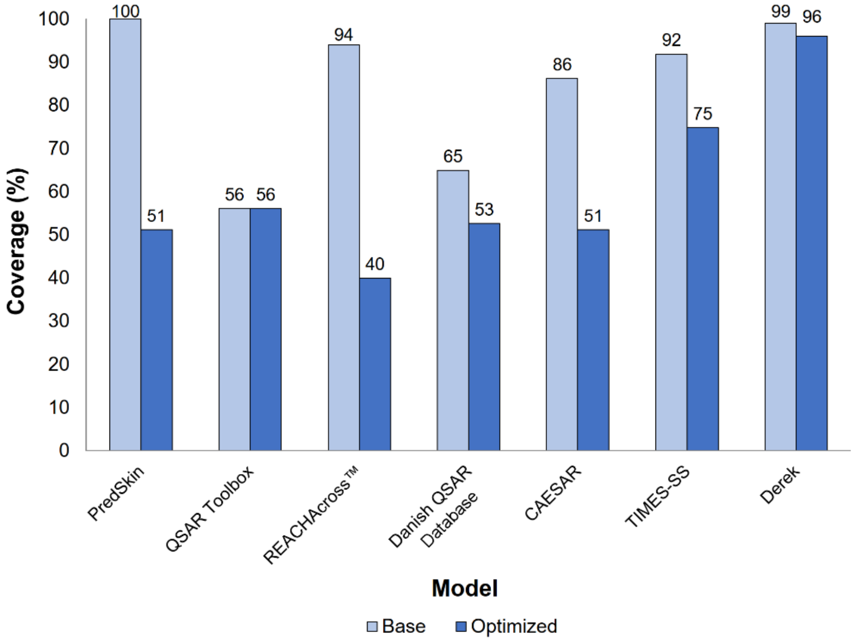

Fig. S1: Model coverage for Basketter et al. (2014) data set using both base and optimized settings

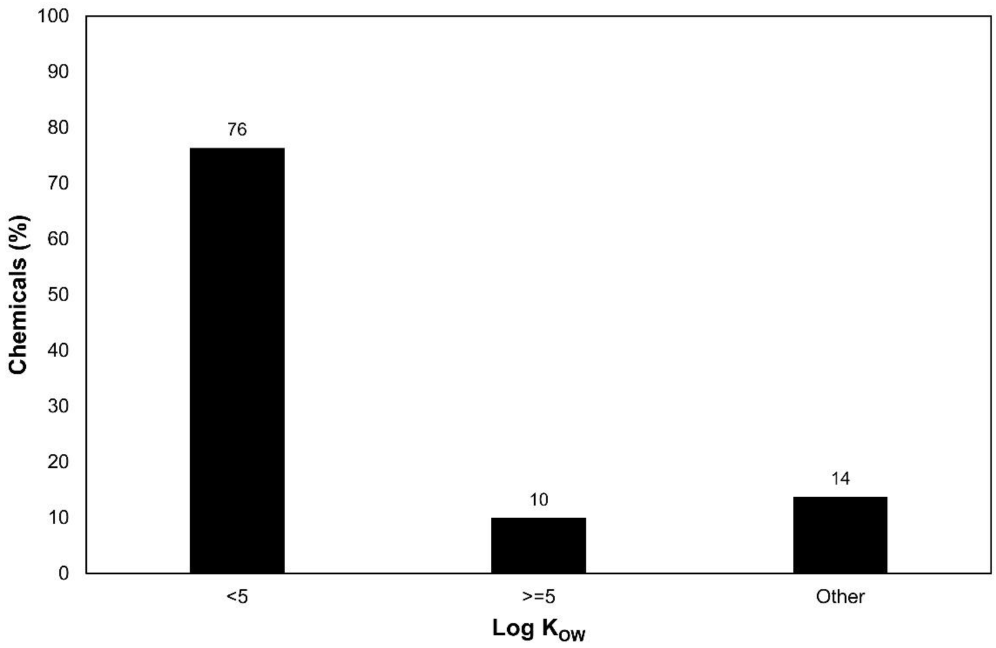

Fig. S2: Partition coefficient distribution for Basketter et al. (2014) data set
"Other" indicates that no experimental data were available, and a prediction could not be made due to a problem with the SMILES string.

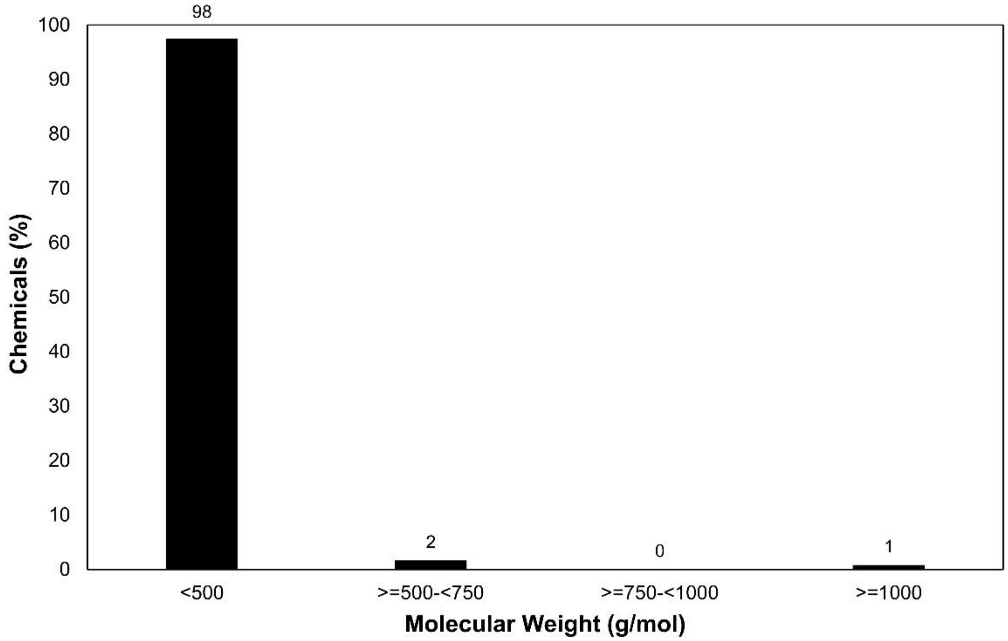

Fig. S3: Molecular weight distribution for Basketter et al. (2014) data set

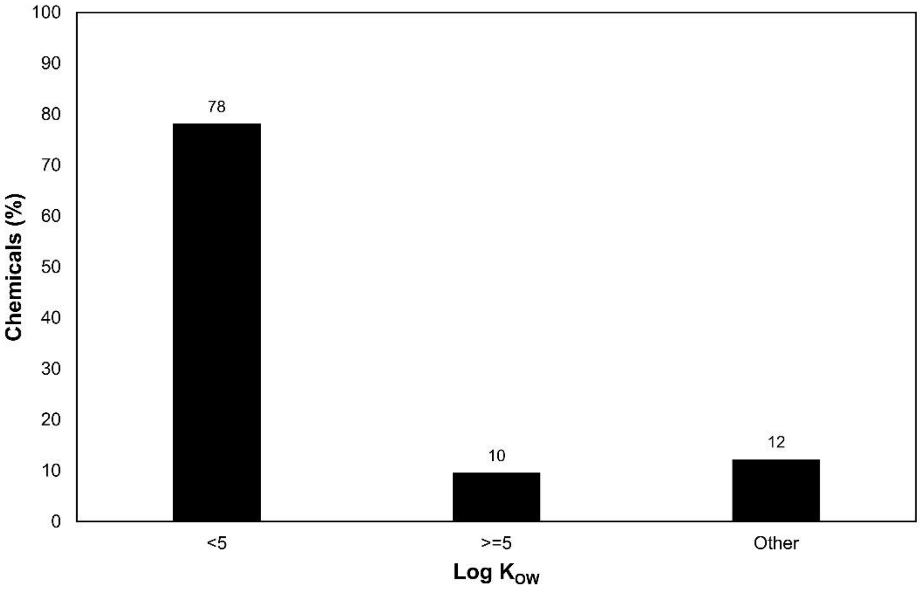

Fig. S4: Partition coefficient distribution for HSDB data set
"Other" indicates that no experimental data were available, and a prediction could not be made due to a problem with the SMILES string.

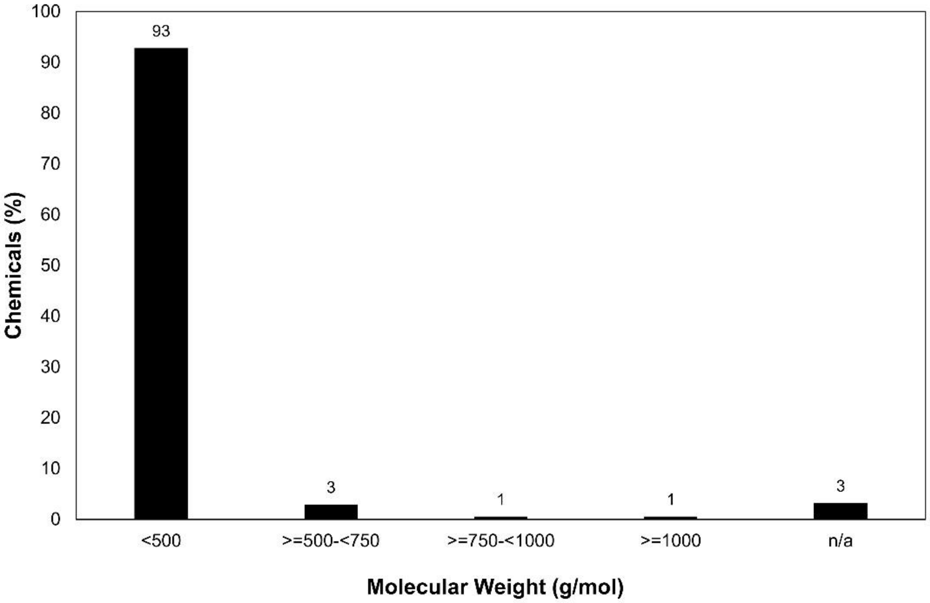

Fig. S5: Molecular weight distribution for HSDB data set
"n/a" indicates that the chemical was a mixture, so no discrete molecular weight could be identified.
